# The homeostasis of β‐alanine is key for Arabidopsis reproductive growth and development

**DOI:** 10.1111/tpj.70134

**Published:** 2025-04-03

**Authors:** Si Wu, Youjun Zhang, Urszula Luzarowska, Lei Yang, Mohamed A. Salem, Venkatesh P. Thirumalaikumar, Nir Sade, Vadim E. Galperin, Alisdair Fernie, Arun Sampathkumar, Shimon Bershtein, Corina M. Fusari, Yariv Brotman

**Affiliations:** ^1^ Department of Life Sciences Ben Gurion University of the Negev Beersheva Israel; ^2^ Max Planck Institute of Molecular Plant Physiology Am Mühlenberg Potsdam‐Golm 114476 Germany; ^3^ Purdue Proteomics Facility Bindley Biosciences, Purdue University West Lafayette Indiana 47907 USA; ^4^ School of Plant Sciences and Food Security Institute for Cereal Crops Research, Tel Aviv University Tel Aviv 69978 Israel; ^5^ BLAVATNIK CENTER for Drug Discovery Tel Aviv University Tel Aviv 69978 Israel; ^6^ Centro de Estudios Fotosintéticos y Bioquímicos (CEFOBI‐CONICET‐UNR) Suipacha 570 Rosario S2000LRJ Argentina; ^7^ Present address: Computational Oncology, AbbVie South San Francisco California 94080 USA

**Keywords:** metabolic genome‐wide association studies, β‐alanine, metabolic regulation, *AGT2*, *ALDH6B2*, plant development, seed abortion, yield traits

## Abstract

β‐Alanine, an abundant non‐proteinogenic amino acid, acts as a precursor for coenzyme A and plays a role in various stress responses. However, a comprehensive understanding of its metabolism in plants remains incomplete. Previous metabolic genome‐wide association studies (mGWAS) identified *ALANINE:GLYOXYLATE AMINOTRANSFERASE2 (AGT2*, AT4G39660) linked to β‐alanine levels in Arabidopsis under normal conditions. In this study, we aimed to deepen our insights into β‐alanine regulation by conducting mGWAS under two contrasting environmental conditions: control (12 h photoperiod, 21°C, 150 μmol m^−2^ sec^−1^) and stress (harvested after 1820 min at 32°C and darkness). We identified two highly significant quantitative trait loci (QTL) for β‐alanine, including the *AGT2* locus associated in both environments and *ALDEHYDE DEHYDROGENASE6B2* (*ALDH6B2*, AT2G14170) associated only under stress conditions. A coexpression‐correlation network revealed that the regulatory pathway involving β‐alanine levels, *AGT2*, and *ALDH6B2* connects the branched chained amino acid (BCAA) degradation through the propionate pathway. Metabolic profiles of *AGT2* overexpression (OE) and knock‐out (KO) lines (*agt2*) across various organs and developmental stages established the critical role of AGT2 in β‐alanine metabolism. This work underscores the importance of β‐alanine homeostasis for proper growth and development in Arabidopsis.

## Significance Statement

Metabolic GWAS revealed that β‐alanine levels are differentially regulated under control and stress conditions by the action of two genes. *AGT2* is robustly associated with both conditions. Its function exceeds the control of β‐alanine levels as *agt2* knock‐out mutants exhibited growth and developmental defects. Moreover, *ALDH6B2* is key in stress conditions, and its function links the regulation of the branched chain amino acid degradation pathway with the β‐alanine pathway.

## INTRODUCTION

β‐Alanine is a non‐proteinogenic amino acid found in all living organisms. In plants and microbes, β‐alanine is the precursor of pantothenic acid (vitamin B_5_). Pantothenic acid is in turn the precursor of coenzyme A, a cofactor required by many metabolic enzymes (White et al., 2001). In addition, β‐alanine is an important stress‐associated metabolite (Cona et al., 2006), serving as a precursor for betaine, an osmo‐protectant found in species of Plumbaginaceae (Hanson et al., 1994; Raman & Rathinasabapathi, 2003). In some legumes, β‐alanine, along with γ‐glutamate and cysteine, forms the thiol tripeptide homoglutathione, an antioxidant involved in heavy‐metal detoxification and in protection against reactive oxygen species (Matamoros et al., 1999; Moran et al., 2000). Moreover, β‐alanine plays a prominent role in lignin biosynthesis as one of the metabolites directly involved in coenzyme A synthesis and as a precursor of many secondary metabolites (Broeckling et al., 2005; Cona et al., 2006).

The three proposed pathways for β‐alanine biosynthesis in plants include uracil degradation, polyamine oxidation, and propionate metabolism. Although there is some evidence for these three pathways (Cona et al., 2006), only one study characterized in detail the last enzyme in the uracil degradation pathway (Walsh et al., 2001). In addition, it has been demonstrated for Arabidopsis and wheat that the branched chain amino acid (BCAA) degradation pathway can supply precursors for β‐alanine synthesis. For instance, isoleucine catabolism ends up in propionyl‐CoA (Gipson et al., 2017). Recently, Goldfarb et al. (2024) used isotopically labeled isoleucine and propionate to prove that the synthesis of β‐alanine is occurring via transamination of malonate semialdehyde. Valine degradation, either through the production of methylmalonate semialdehyde or via amino acid homeostasis, also contributes to increasing levels of β‐alanine (Perrett et al., 2018). Although absent in eukaryotes, decarboxylation of l‐aspartate is the major pathway for β‐alanine biosynthesis in prokaryotes (Cronan, 1980; Wang et al., 2014).

Considerable insight into the biosynthesis of β‐alanine has been obtained in plants, and this is comprehensively reviewed by Parthasarathy, Savka, and Hudson (2019). For example, for Arabidopsis, genes encoding enzyme activities involved in the uracil and polyamine pathway have been described (eight genes for seven enzymatic reactions, KEGG β ‐alanine pathway in Arabidopsis). However, 60% of the enzymatic reactions involved in the remaining pathways of β‐alanine metabolism have not been assigned to any gene. One of the reactions not yet assigned to any specific gene is the degradation of β‐alanine via the transamination of pyruvate or other α‐keto‐acids. This process is catalyzed by a β‐alanine‐α‐keto‐acid aminotransferase (Parthasarathy, Savka, & Hudson, 2019). Due to the molecular similarities between l‐alanine and β‐alanine, transaminases may act on either amino acid as a donor of the amino group. Based on sequence homology, three genes encoding these enzyme activities have been systematically annotated as alanine:glyoxylate aminotransferases in Arabidopsis: AT2G38400 (*AGT3*), AT3G08860 (*PYD4*) and AT4G39660 (*AGT2*). To date, only *PYD4* has been confirmed as l‐alanine:glyoxylate aminotransferase but not as β‐alanine aminotransferase (Parthasarathy, Adams, et al., 2019).

Our previous study using natural variation showed a strong association between β‐alanine levels and *AGT2* (AT4G39660) in normal growth conditions, and a significant increase in β‐alanine levels in leaves for two independent *agt2* knock‐out (KO) lines (Wu et al., 2016). In maize, Wen et al. (2015) reported ZM01G05170 as being associated with β‐alanine levels. A phylogenetic analysis showed that *AGT2* and ZM01G05170 clustered together and separately from *AGT3* and *PYD4*, suggesting that this gene family underwent functional differentiation prior to speciation, followed by subsequent gene duplication within species (Wu et al., 2016). In this context, *AGT2* emerges as the most likely candidate for β‐alanine‐aminotransferase function in Arabidopsis.

In this work, we conducted metabolic genome wide association studies (mGWAS) for β‐alanine levels in two contrasting environmental conditions. We grew the population in optimal growth conditions (i.e., control, 21‐L: 16 h light photoperiod and 150 μmol m^−2^ sec^−1^) or we subjected the population to a sudden environmental change (i.e., stress, 32‐D: 1280 min of darkness and 32°C before harvesting). We found two highly significant QTL for β‐alanine levels: *AGT2* locus (AT4G39660) associated in both environments, and *ALDH6B2* (AT2G14170) associated only in stress. By employing co‐expression‐correlation networks, we further unraveled the regulatory pathway involving *AGT2* and *ALDH6B2*. This analysis enabled us to understand the connection between BCAA degradation and β‐alanine metabolism. Metabolic profiles in *AGT2* overexpression (OE) and KO lines (*agt2*) together with metabolic analysis of *agt3* and *pyd4 mutants*, demonstrated that *AGT2* is a key player in β‐alanine metabolism. Finally, this work provides clear evidence that fine‐tuning of β‐alanine levels plays a pivotal role in growth and development in Arabidopsis.

## RESULTS

### mGWAS of β‐alanine in two contrasting environmental setups

In previous work of our group, mGWAS showed a strong association between β‐alanine and *AGT2* in control conditions, and we further validated the impact of *AGT2* on β‐alanine levels using two independent KO lines (Wu et al., 2016). We also found that β‐alanine increases dramatically under different light and temperature conditions (Caldana et al., 2011). This is in line with several studies reporting that β‐alanine is highly involved in response to abiotic stresses (Parthasarathy, Savka, & Hudson, 2019). To further identify what genes are involved in the regulation of β‐alanine levels under sudden environmental fluctuations, we grew a natural Arabidopsis population in control (21‐L, 16 h light photoperiod and 150 μmol m^−2^ sec^−1^) and stress (32‐D: 1280 min of darkness and 32°C before harvesting) and performed mGWAS (Data S1).

**Figure 1:**
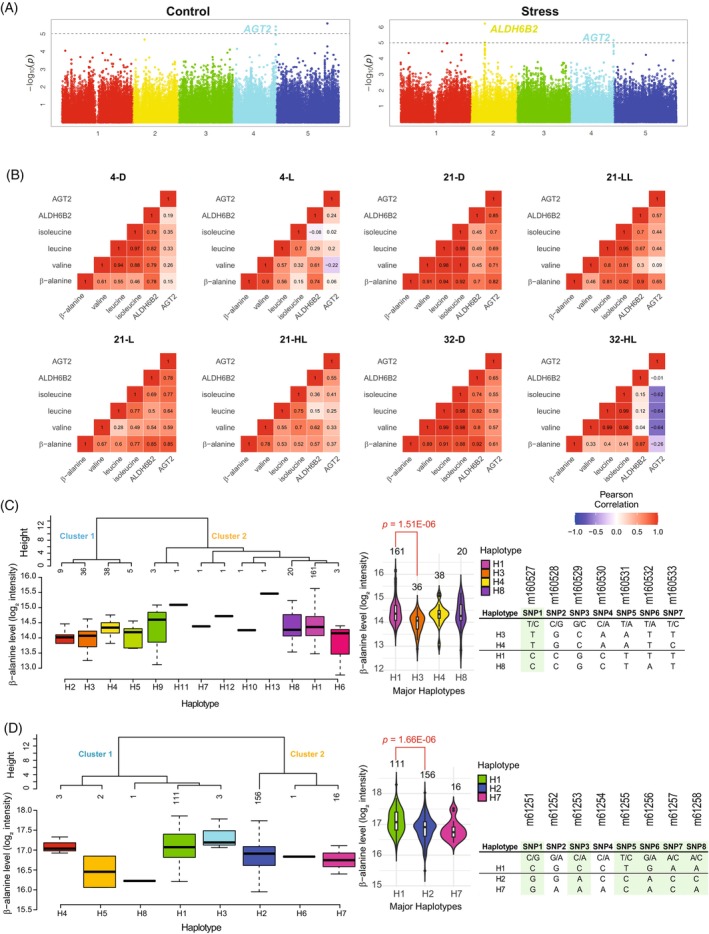
Natural variation for β‐alanine levels from plants grown under control (CHD) and heat and darkness (HD) conditions. (A) Manhattan plots obtained for β‐alanine levels (log_2_ of intensity) under control (CHD, left) and under stress (HD, right). The *AGT2* (AT4G39660) QTL was found at Chromosome 4 under both conditions. The QTL *ALDH6B2* (AT2G14170) was detected at Chromosome 2 only under HD conditions. (B) Pearson correlation coefficients calculated between β‐alanine levels, AGT2 and ALDH6B2 expression and branched‐chain amino acids (valine, leucine and isoleucine) for Col‐0 ecotype across eight different conditions. (C) Haplotype analysis using the seven SNPs in *AGT2* coding region, scored in the SNPChip. Clusters were obtained using Ward's distance on pairwise dissimilarity. Average trait value (β‐alanine intensity, log_2_ scale) for the different *AGT2* haplotypes, 2^(−ΔΔCt)^ minor allele frequency of 5%. Table of alleles for the haplotypes plotted. The putative causal SNP is highlighted in green. (D) Haplotype analysis using the eight SNPs in *ADLH6B2* coding region, scored in the SNPChip. Clusters were obtained using Ward's distance on pairwise dissimilarity. Average trait value (β‐alanine intensity, log_2_ scale) for the different *ALDH6B2* haplotypes, minor allele frequency of 5%. Table of alleles for the haplotypes plotted. The putative causal SNPs are highlighted in green.

As shown in Figure 1(A), β‐alanine levels mapped to *AGT2* in both control and stress conditions with LOD values 5.4 and 5.2, respectively [LOD = −log_10_(*P*‐value); Logarithm of Odds]. We were able to reproduce our previous findings from control conditions (Wu et al., 2016), indicating that *AGT2* is one of the key genes controlling β‐alanine levels. In addition, the association with *AGT2* is robust enough to be detected in several environmental setups. On the other hand, we observed that β‐alanine levels mapped to a locus located in Chromosome 2 only in the stress condition. This locus harbors the candidate gene *ALDH6B2* (AT2G14170), a methylmalonate‐semialdehyde dehydrogenase. *ALDH6B2* is thought to be involved in the generation of propionyl‐CoA from methylmalonate‐semialdehyde during valine degradation or to produce acetyl‐CoA from malonate‐semialdehyde. Both compounds, propionyl‐CoA and malonate‐semialdehyde, are involved in the β‐alanine biosynthetic pathway (Gipson et al., 2017).

Because BCAAs have been proposed to be involved in β‐alanine biosynthesis (Gipson et al., 2017), we further assayed mGWAS for valine, leucine, and isoleucine levels in control and stress conditions. We did not detect associations either between BCAA and *AGT2* locus or between BCAAs and *ALDH6B2* locus (Figure S1). We next checked the correlations between β‐alanine, BCAA, *AGT2*, and *ALDH6B2* in eight different environmental conditions for Col‐0 (Caldana et al., 2011); see full description in “Experimental procedure” section. In general, we observed a comprehensively high positive correlation between these components, indicating that they may constitute a tightly connected regulatory network (Figure 1B). BCAA members share high positive correlations between each other in most of the eight conditions. As we already reported (Wu et al., 2016), β‐alanine and *AGT2* transcript had significant positive correlations in four different conditions (32‐D, 21‐D, 21‐LL and 21‐L, *r* ^2^ > 0.6, *P* < 0.01). Moreover, we also observed a high positive correlation between β‐alanine and *ALDH6B2* transcript in 32‐D condition (*r* ^2^ = 0.92, *P* < 0.01), going along with the association found in the mGWAS between β‐alanine levels and *ALDH6B*2. We also found a robust correlation between β‐alanine and BCAA, particularly in dark conditions. This is in close agreement with other studies showing that β‐alanine can be generated through isoleucine directly and valine indirectly, respectively (Goldfarb et al., 2024; Parthasarathy, Adams, et al., 2019). The correlation between *AGT2* and *ALDH6B2* was only significant at 21‐D (*r* ^2^ = 0.85, *P* < 0.001). The lack of correlation for the remaining conditions is consistent with the fact that there was no epistatic interaction between *AGT2* and *ALDH6B2* loci (*P* = 0.346; Figure S2). These results could imply that both genes regulate β‐alanine levels through independent mechanisms at least under the conditions tested for mGWAS here.

To evaluate causal mutations in *AGT2*, we performed haplotype analysis. Using SNPchip information, we found seven SNPs in the *AGT2* gene region, giving rise to 13 possible haplotypes (Figure 1C). Eight of them were informative haplotypes (i.e., defined by more than two accessions per haplotype) and four have an allele frequency higher than 5% (i.e., minor allele frequency [MAF]‐used for GWAS). According to haplotype sequence similarity, they could be further grouped into two main clusters: Cluster 1 (H2, H3, H4, and H5) and Cluster 2 (H1, H6–H13). In addition, β‐alanine mean values for haplotypes with ≥5% MAF were significantly different (one‐way anova, *P*‐value = 5.98E‐06). The most significant pairwise difference was found between H1 and H3, two haplotypes in different clusters and with opposite alleles for six out of the seven SNPs in the haplotype. Further analysis on the type of polymorphism revealed that SNP1 (m160527) is a non‐synonymous change that could explain the underlying association between β‐alanine levels and *AGT2* (Table S1).

In *ALDH6B2*, sequence analysis revealed eight SNPs, combining into eight different haplotypes. These haplotypes could be further grouped into two main clusters: Cluster 1 (H1, H4, H5, H8) and Cluster 2 (H2, H6, H7) (Figure 1D). We detected significant differences in β‐alanine levels comparing haplotypes with ≥5% MAF (one‐way anova, *P*‐value = 1.46E‐06). The most significant pairwise difference was found between H1 and H2, the two major haplotypes for *ALDH6B2*. They group in different clusters and displayed opposite alleles for six out of the eight SNPs. The putative causal polymorphism was either synonymous or silent (Table S1), suggesting that this gene may regulate β‐alanine levels at the transcriptional/post‐transcriptional level. Alternatively, these SNPs might be in linkage disequilibrium with additional, unscored non‐synonymous SNPs in the gene.

### Transcriptional regulation of AGT2

#### eQTL studies

**Figure 2:**
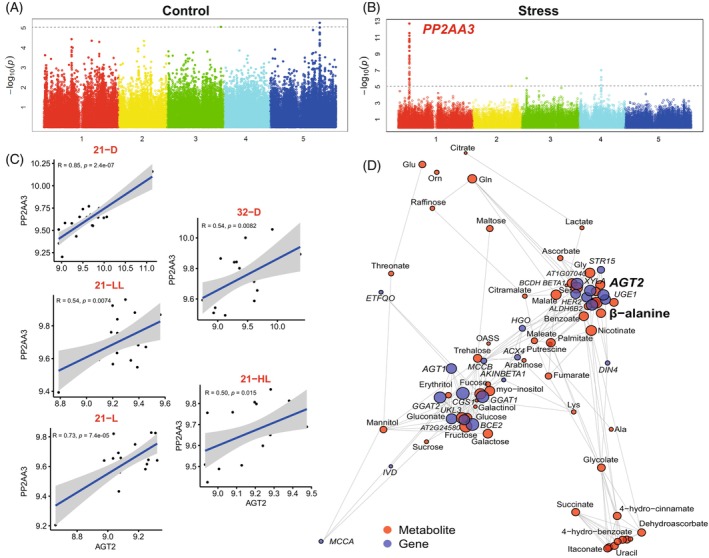
Transcriptional regulation of AGT2. Manhattan plots obtained for *AGT2* expression levels (2^(−ΔΔCt)^) under control conditions (A) and under stress (B). The *PP2AA3* (AT1G3320) QTL was found at Chromosome 1 under stress. The threshold of association (LOD = 5) is marked with a dash line. (C) Correlations calculated between *AGT2* and *PP2AA3* expression for Col‐0 ecotype across eight different conditions. (D) Coexpression‐correlation network using 28 genes (blue) coexpressed with *AGT2* in eight different environmental conditions, and 54 significantly changed primary metabolites (red) in 21‐L condition. Size of symbols represents the degree of correlation with *AGT2*.

To investigate the transcriptional regulatory network underlying *AGT2*, we conducted GWAS for *AGT2* expression levels from the complete Arabidopsis panel grown in control (21‐L) and stress (32‐D) conditions (Data S2). In control conditions, *AGT2* expression showed a peak of moderate association (LOD = 6.03) in Chromosome 5 (Figure 2A). Functional annotations for candidate genes in the region are not *a priori* related to regulation of expression. For the population subjected to stress (1280 min of 32°C and darkness), we detected a highly significant association (LOD = 12.74) between *AGT2* expression and a locus in Chromosome 1 (Figure 2B). This locus harbors the gene *PP2AA3* (AT1G3320), which is a protein phosphatase 2A subunit A3. It is reported that protein phosphatase 2A is a regulator of ROS signaling in plants, facilitating plants to acclimate to abiotic (e.g., varying light conditions) and biotic stresses (Konert et al., 2014; Rahikainen et al., 2016). Therefore, we also investigated the correlation between *AGT2* and *PP2AA3* in the eight conditions differing in light and temperature. We observed that *AGT2* and *PP2AA3* expression levels are significantly and positively correlated in five different conditions (21‐D, 21‐LL, 21‐L, 21‐HL 32‐D, 32‐L) (Figure 2C).

#### Co‐expression‐correlation network

To further understand the biological processes involving *AGT2*, we analyzed 28 *AGT2* co‐expressed genes (Table S2) based on data from two public databases: ATTED‐II: https://atted.jp/ and STRING: https://string‐db.org/. First, we calculated the correlations between *AGT2* and the co‐expressed genes by using our time‐series expression data across eight different conditions (Caldana et al., 2011). Most of the co‐expressed genes showed a significant correlation with *AGT2* in the 32‐D, 21‐D, 21‐L, and 21‐LL conditions, the same conditions where β‐alanine displayed a significant positive correlation with *AGT2* (Figure S3).

The co‐expressed genes positively correlating with *AGT2* are involved in BCAA degradation, the propionate pathway and the uracil catabolic pathway. These three pathways provide precursors for β‐alanine synthesis. Additionally, another set of genes positively correlating with *AGT2* are described as being involved in β‐alanine metabolism, further supporting *AGT2*'s role within this pathway. Conversely, enzymes involved in the synthesis of proteinogenic amino acids, glycine/serine/threonine metabolism or alanine/aspartate/glutamate metabolism showed a significant negative correlation with *AGT2*.

Finally, we combined the co‐expressed genes with the significantly altered primary metabolites under normal condition (21‐L) to construct a co‐expression‐correlation network (Figure 2D). A prominent cluster in the upper‐right part of the network includes β‐alanine and *AGT2* as its center, alongside amino acids (glutamate, glutamine, serine), TCA cycle intermediates (malate, fumarate, citrate), and genes involved in BCAA degradation (e.g., *ALDH6B2*, *UGE1*: AT1G12780, *BCDH‐BETA1*: AT1G55510). This cluster is closely connected to another in the bottom‐left, which primarily contains sugars (e.g., maltose, galactose, glucose, trehalose, sucrose) and genes involved in amino acid metabolism (*GGAT1*: AT1G23310, *AGT1*: AT2G13360, *GGAT2*: AT1G70580).

Notably, two genes related to plant's carbon status, *AtKINBETA1* (AT5G21170) and *DIN1* (AT4G35770), are co‐expressed with *AGT2*. These connections suggest that β‐alanine may be a key metabolite in the energy‐homeostasis signaling network, sensed by SnRK1 (Blanco et al., 2019) and *DIN* genes (Fujiki et al., 2000).

### AGT2 is a key gene involved in β‐alanine metabolism in Arabidopsis

In Arabidopsis, there are two other close homologs to *AGT2*: *AGT3* (AT2G38400) and *PYD4* (AT3G08860). Based on publicly available expression data (eFP Browser, http://bar.utoronto.ca/efp/cgi‐bin/efpWeb.cgi) (Sullivan et al., 2019), *AGT2* is constitutively expressed in most organs, with increasing expression in seeds, roots, and stems. In contrast, *PYD4* and *AGT3* show generally low expression, except in later stages of seed development, mature pollen, and senescent leaves.

To investigate whether these genes influence β‐alanine levels, we analyzed two *agt2* KO lines (SALK_003381: *agt2_1*; SALK_035035: *agt2_2*) three *agt3* KO lines (SALK_100364, SALK_146687, SALK_144859) and four *pyd4* KO lines (SAIL_400_G02, SALK_002102, SALK_001698, SALK_141570). In addition, three independent constitutive OE lines of *AGT2* were generated.

We conducted targeted metabolomics in rosettes and mature seeds of wild‐type Col‐0, along with the *agt2*, *agt3*, and *pyd4* knockout (KO) lines, as well as the *AGT2* overexpression (OE) lines (Tables [Link], [Link], [Link], [Link], [Link], [Link]). We profiled between 78 and 94 primary metabolites (i.e., carbohydrates, organic acids and amino acids) that changed significantly in the KO and/or OE lines compared to Col‐0.

**Figure 3:**
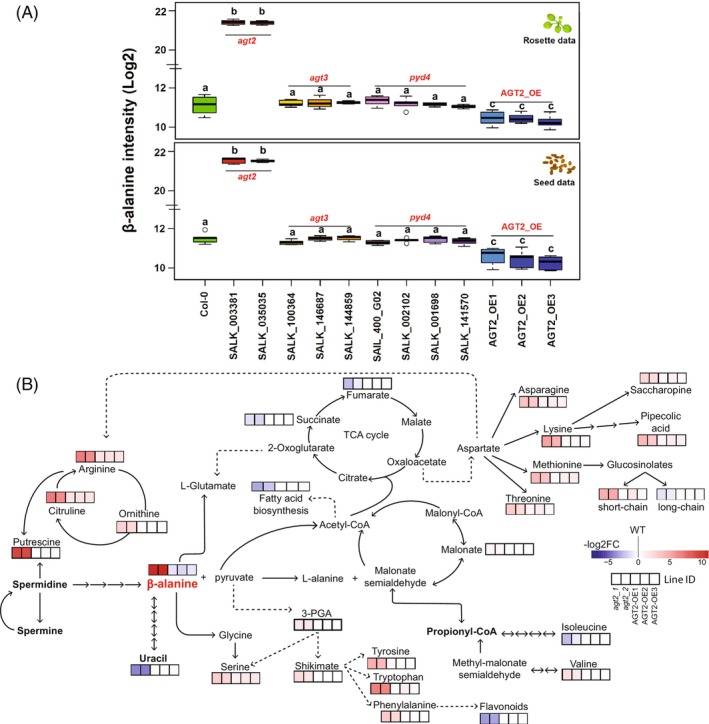
Metabolic changes in *agt2*‐KO and *AGT2*‐OE lines. (A) β‐alanine levels (log_2_ of intensity) for *agt2* (SALK_003381, SALK_035035), *AGT2*‐OE, *agt3* (SALK_100364, SALK_146687, SALK_144859) and *pyd* *4* (SAIL_400_G02, SALK_002102, SALK_001698, SALK_14570), measured in rosettes and seeds. Wild‐type Col‐0 was used as reference. Statistical analysis: one‐way anova followed by Tukey *post hoc* test. Means (*n* = 5) with distinct letters indicate significant differences (*P*  < 0.05). (B) Main significant metabolic changes observed in seeds from *agt2* ‐KO (SALK_003381, SALK_035035) and three *AGT2*‐OE lines. Heatmap values are log_2_ fold change of metabolite level, compared with wild‐type Col‐0. Main precursors of β‐alanine biosynthesis are shown in bold (spermidine, spermine, uracil and propionyl‐CoA). Dashed arrows simplified enzymatic reactions and intermediates not specified here. The three main products of β‐alanine degradation described *in vivo* or tested *in vitro* (Figure S5) are included here (l‐alanine, l‐glutamate, and glycine). Complete heatmap for primary and secondary metabolites, and lipids can be found in Figure S7, metabolite levels and statistical analyses are in Tables S7 and S8.

In both organs (seeds and leaves), *agt2* KO lines showed significantly increased levels of β‐alanine, while *AGT2* OE lines exhibited a significant decrease in β‐alanine levels. The β‐alanine levels in *agt3* and *pyd4* KO lines remained constant and like those in Col‐0 (Figure 3A). Analysis of overall primary metabolic changes in *agt3* and *pyd4* mutants revealed that several metabolites decreased significantly compared to Col‐0, with more metabolites showing significant changes in seeds than in rosette samples (Figure S4). This is consistent with the higher expression levels of *AGT3* and *PYD4* in seeds compared to rosettes. Among the main metabolic pathways affected in these mutants, amino acids, organic acids, and carbohydrate metabolism are the most impacted. Specifically, both rosette and seed samples exhibited significant changes in amino acids such as alanine, leucine, histidine, phenylalanine, tyrosine, and proline. These results confirm that amino acid turnover is altered in these mutants, although the β‐alanine pathway remains unaffected.

These results reinforced the idea that AGT2 alone accounts for β‐alanine level variation in Arabidopsis and functions primarily in the direction of β‐alanine degradation, while AGT3 and PYD4 are involved in different enzymatic reactions (most probably in alanine transamination). To test this hypothesis, we selected the sequences of the two major alleles (strong and weak) responsible for the high and low levels of β‐alanine in the GWA population, respectively. The sequences for *AGT2_strong* and *AGT2_weak* alleles were obtained from Col‐0 and C24 re‐sequenced information, respectively (Data S3). We also include Col‐0‐*PYD4* as a positive control for alanine aminotransferase activity (Parthasarathy, Adams, et al., 2019). We were able to purify the recombinant proteins *AGT2_weak* and Col‐0‐*PYD‐4* from the soluble fraction. However, *AGT2_strong* was retained in the precipitate. Therefore, we could not test enzyme activity with the *AGT2_strong* allele.

We test if AGT2 can use β‐alanine as an amino donor, with either pyruvate, glyoxylate, or 2‐oxoglutarate as amino acceptors *in vitro* (Figure S5). These reactions would yield 3‐oxopropanoate along with l‐alanine, glycine, or glutamate, respectively. However, we did not detect the formation of l‐alanine when using pyruvate as an amino acceptor (Figure S5c). Furthermore, although we observed pyruvate dehydrogenase activity as a coupled enzyme when using glyoxylate or 2‐oxoglutarate as amino acceptors, we could not directly detect the formation of 3‐oxopropanoate Therefore, we cannot conclude with certainty that AGT2 functions as a β‐alanine aminotransferase (Figure S5a,b).

We also detected activity when l‐alanine was used as the amino donor, but unlike PYD4, AGT2 only functions in the direction of degradation (Figure S5d–g). Contrary to previous reports (Parthasarathy, Adams, et al., 2019), and in agreement with a recent publication, PYD4 was also able to use β‐alanine as an amino donor (Goldfarb et al., 2024).

### The influence of *AGT2* on β‐alanine homeostasis triggers tissue‐differential metabolic reprogramming

We extended our analyses of primary metabolites in *agt2* to seven different organs: seed, seedling, rosette, stem, flower, senescent leaf, and silique. Most metabolites were detected in all organs (48 out of 71, 67.7%) (Figure S6a; Table S9). Among metabolites that changed in both *agt2* lines compared to the wild type (Figure S6b; Table S10), β‐alanine was the only metabolite that was significantly upregulated in all seven organs (Figure S6c). This finding suggests that *AGT2* is crucial for maintaining β‐alanine levels within specific ranges across different organs and developmental stages.

We conducted pathway enrichment analysis on the 62 primary metabolites that were significantly different in at least two organs (Figure S7). The significantly enriched pathways included glycine, serine and threonine metabolism, arginine biosynthesis, cysteine and methionine metabolism, as well as alanine, aspartate and glutamate metabolism (FDR < 0.05, impact scores >0.2). Many transcripts from enzymes involved in the synthesis or degradation of those amino acids showed a significant negative correlation with *AGT2* in control conditions (Figure S3). Together, these findings suggest that *AGT2*, and consequently β‐alanine levels, have a direct effect on amino acid metabolism.

In addition, we extended the metabolic profiling to 20 secondary metabolites (i.e., flavonoids and glucosinolates) and 133 lipids (i.e., phosphatidylethanolamine, phosphatidylcholine, diacylglycerols, and triacylglycerol) in seeds of *agt2* KO and *AGT2*OE lines (Tables S7 and S8). As reported above, β‐alanine showed significant accumulation in the *agt2* KO lines and a significant decrease in the OE lines, respectively. Knocking out *AGT2* led to a significantly higher amount of putrescine and a significantly lower level of uracil. Both the polyamine and the pyrimidine pathways provide precursors for β‐alanine synthesis (Figure 3B; Figure S8; Tables S7 and S8). Moreover, *agt2* mutants displayed significantly increased levels of several amino acids, including the shikimate‐derived aromatic amino acids and the urea‐related amino acids (Figure 3B; Figure S9; Tables S7 and S8). Overall, amino acid metabolism is reprogrammed, impacting specific catabolic pathways.

In contrast, overexpressing *AGT2* had a mild effect on the metabolome. Besides a few other amino acids that increased significantly (serine, asparagine and arginine), only β‐alanine showed a significant decrease in the OE lines.

Regarding secondary metabolites, we found that flavonoids were significantly decreased, while glucosinolate levels exhibited bidirectional changes in the *agt2* KO lines. For instance, short‐chain glucosinolates increased, whereas long‐chain glucosinolates decreased (Figure 3B; Figure S8; Tables S7 and S8).

For major lipid‐based carbon storage in triacylglycerols (TAGs) as well as for structural lipids, we observed a global downregulation, with 53 lipid species significantly decreased (Figure S8; Tables S7 and S8).

### Plant growth and development are affected by β‐alanine levels

**Figure 4:**
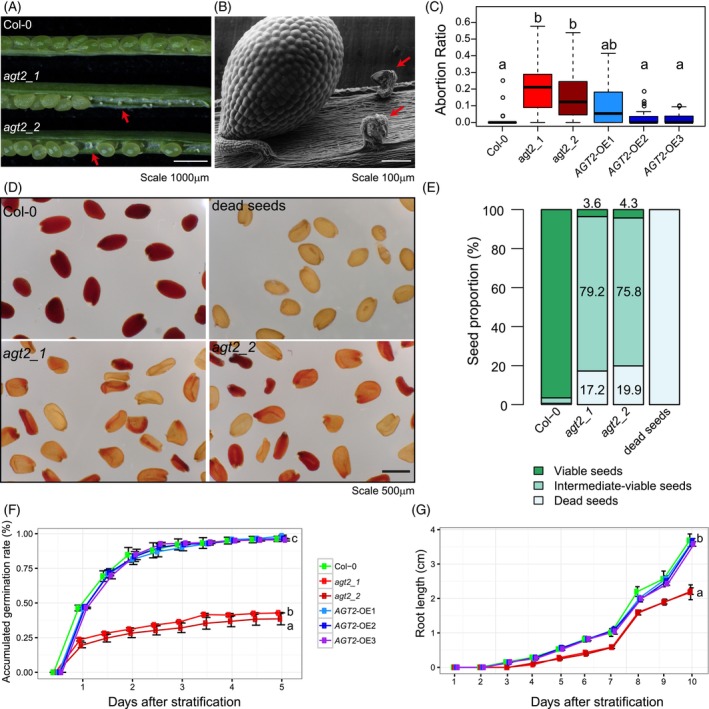
AGT2 function in development. (A) Seed‐abortion phenotype observed in siliques from *agt2_1* and *agt2_2* mutants, compared with siliques from wild‐type Col‐0 (Light stereo microscope). Red arrows show the funiculus without seed. Scale included in the figure. (B) Scanning Electron Microscopy (SEM) of a silique from *agt2_1* (SALK_003381). Red arrows show the funiculus. (C) Abortion ratio (number of aborted seeds/number of total seeds; 40 silique per plant; five to six plants per line) in wild type, *agt2* KO lines, and *AGT2*‐OE lines. Significant differences were detected using one‐way anova followed by Tukey *post hoc* test. Means with distinct letters indicate significant differences (*P* < 0.05). (D) Seed viability using tetrazolium assay for wild‐type Col‐0, *agt2* KO mutants freshly harvested dry mature seeds, and dead seeds (Col‐0 freshly harvested seeds incubated at 100°C for 1 h). (E) Proportion of viable seeds, intermediate‐viable seeds and dead seeds according to the color of staining, for the same lines as in (D). (F) Germination rate for wild‐type Col‐0, *agt2* KO lines, and *AGT2*‐OE lines after stratification (*n* ~100 seeds from three to four different plants per line) register up to 10 days after stratification. Two‐way anova followed by a Tukey *post hoc* test was used to detect significant differences between KO and the remaining lines (different letters) across all curve‐points (*P* < 0.05). (G) Root length measurements for wild‐type Col‐0, *agt2* KO lines, and *AGT2*‐OE lines after stratification (*n* = 8–12 plants per line) register up to 10 days after stratification of seeds. Two‐way anova followed by a Tukey *post hoc* test was used to detect significant differences between KO and the remaining lines (different letters) across all curve‐points (*P* < 0.05).

The two *agt2* lines showed a low germination rate (Table S11). To investigate the cause of this phenotype, we first examined the general morphology and anatomical features of seeds in the *agt2* and wild‐type Col‐0 lines. The *agt2* lines exhibit severe seed abortion (Figure 4). Additionally, seeds in both mutants were detached from the funiculus, the narrow green strand that anchors seeds to the maternal plant (Figure 4A,B). Quantitatively, both *agt2* lines show a significantly higher abortion ratio compared to Col‐0, while the OE lines had a lower abortion ratio and a phenotype more similar to the wild type (Figure 4C; Table S12).

Next, we assessed seed viability using a tetrazolium‐based assay (Verma & Majee, 2013). Living seeds absorb the colorless tetrazolium reagent and convert it into formazan, resulting in a brown‐reddish color. Seeds of both *agt2* lines show pale staining compared to wild type, indicating lower seed viability (Figure 4D; Figure S8). We classified seeds into three groups based on their staining intensity: viable seeds (dark brown/reddish), intermediately viable seeds (light brown) and dead seeds (yellowish). We then quantified them across different genotypes. For *agt2* KO lines, approximately 18% of seeds were dead, around 4% were viable, and the rest displayed intermediate viability (Figure 4D,E; Table S13). The reduced seed viability corresponded with a lower germination rate in both *agt2* lines, while the three OE lines maintained a germination rate similar to the wild type (Figure 4F; Table S11). Tracking root length for 10 days after stratification, KO lines exhibited stunted roots, and OE lines had root lengths similar to that of Col‐0 (Figure 4G; Table S14).

**Figure 5:**
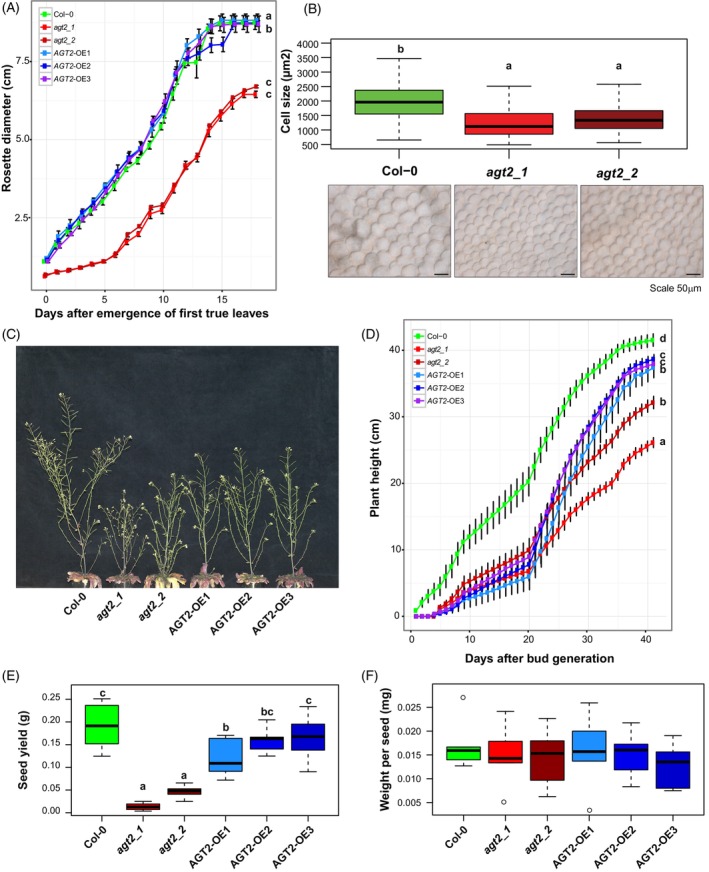
AGT2 function in growth and yield. (A) Rosette diameter for wild‐type Col‐0, two *agt2* KO lines (*agt2_1*: SALK_003381, *agt2_2*: SALK_035035) and three *AGT2*‐OE lines. Rosettes (*n* = 4) for each line were measured every two days from day 0 until 36 days after emergence of first true leaves. A two‐way anova followed by a Tukey *post hoc* test was used to detect differences between lines along the complete curve. Means with distinct letter indicate significant differences (*P* < 0.05). (B) Cell size was scored for the wild‐type Col‐0 and the two *agt2* KO lines (10 cells in three leaves from four plants each line). Significant differences (*P* < 0.05) were detected using one‐way anova followed by Tukey *post hoc* test. Microscopy images from representative leaf fields of wild‐type Col‐0, *agt2_1*, and *agt2_2* KO lines. Scale is shown in the figure. (C) Growth phenotype of wild‐type Col‐0, *agt2* KO lines, and *AGT2*‐OE. (D) Plant height for lines shown in (C). One‐way anova followed by Tukey *post hoc* test (*n* = 10–12 plants per line, *P* < 0.05). (E) Seed yield for each line. Average from 10 plants. Significant differences (*P* < 0.05) were detected using one‐way anova followed by Tukey *post hoc* test. (F) Weight per seed for wild‐type Col‐0, *agt2* KO lines, and *AGT2*‐OE lines. Seeds were collected and weighted from 10 plants each line.

To further investigate the importance of β‐alanine homeostasis in plant growth, we followed the development of *agt2*, OE, and wild type plants from seed germination through 45 days after bud formation. Both *agt2* KO lines, but not OE lines, exhibited a severe reduction in rosette sizes (Figure 5A; Table S15). After the emergence of true leaves, rosettes expanded continuously and rapidly in wild type and OE lines, but lagged in the *agt2* lines in the first 4 days. The gap in rosette sizes persisted in the following days (Figure 5A; Table S15). Using a light microscope, we measured cell sizes and counted cell numbers in a defined area, finding that both *agt2* lines had smaller cells compared to Col‐0 (Figure 5B; Table S16).

At a later developmental stage, when plant growth plateaued, both KO and OE lines were smaller than the wild type (Figure 5C). Bud formation was delayed by 3–4 days in both *agt2* and OE lines (Figure 5D; Table S17). Tracking plant growth after bud formation, we noticed that both KO and OE lines exhibited slower stem growth compared to Col‐0 (Figure 5D; Table S17). During the mid phase of stem growth, the three OE lines surpassed the *agt2* lines in height but still did not reach the height of Col‐0. The final height of KO and OE was about 70% and 80% of Col‐0, respectively.

In addition, we quantified lower seed yield in *agt2* compared to wild type (Figure 5E; Table S18). However, seed weight was not affected among different genotypes, suggesting that the seed yield in the mutants is due to a reduced total number of seeds rather than changes in seed size (Figure 5F; Table S19).

## DISCUSSION

β‐Alanine is an important non‐proteinogenic amino acid found in all organisms. In bacteria and yeast, mutants deficient in β‐alanine biosynthesis are lethal (Cronan, 1980; Merkel & Nichols, 1996; White et al., 2001), probably because β‐alanine is the precursor of pantothenate (vitamin B_5_), which is essential in the production of CoA. In plants, β‐alanine is found to increase in response to abiotic stress. For instance, in cowpea cell cultures, β‐alanine levels were increased two‐fold within 4 h and more than five‐fold after 24 h of heat shock (Mayer et al., 1990). In Arabidopsis, β‐alanine levels were significantly increased when plants were exposed to either heat and/or drought stress (Kaplan et al., 2004; Rizhsky et al., 2004). In our previous studies, Arabidopsis was exposed to a range of environmental conditions, varying in both light intensity and temperature, over a continuous period of 1280 min. Notably, we observed substantial increases in β‐alanine levels in all three conditions associated with darkness, and this trend was consistent across different temperature settings (Caldana et al., 2011; Wu et al., 2016). This combined evidence underscores the impact of carbon limitation on β‐alanine metabolism and strongly suggests that β‐alanine may play a critical role in responding to environmental shifts and fluctuating conditions. In this regard, GWAS has been already used to provide key genetic checkpoints involved in maintaining metabolic homeostasis. For instance, *UGP1* (AT3G02350) and *VAC‐INV* (AT1G12240) associated with central enzyme activities involved in sucrose metabolism, in various environments (Fusari et al., 2017). By contrast, the variation of triacylglycerol levels is associated with *KCS4* (AT1G19440) only under stress (Luzarowska et al., 2023; Zhu et al., 2022). The variation of β‐alanine levels relies on two genes with differential effects: *AGT2* acts irrespective of the environment, and *ALDH6B2* seems to supply further regulation under adverse environmental conditions (Figure 1). In fact, epistasis analysis between both loci revealed that there is an additive genetic effect under stress conditions between *AGT2* and *ALDH6B2* (Figure S2).

Based on sequence homology, *AGT2*, along with *PYD4* and *AGT3*, was annotated as a putative alanine:glyoxylate aminotransferase. However, the specificity of these genes as β‐alanine transaminases has remained unclear (Liepman & Olsen, 2001; Liepman & Olsen, 2003). Notably, Liepman and Olsen (2003) attempted to use alanine as a substrate in enzymatic assays with *AGT2* but concluded that the enzyme is not functional as an alanine aminotransferase. More recent attempts confirmed alanine‐glyoxylate aminotransferase activity (Parthasarathy, Adams, et al., 2019) and β‐alanine transaminase activity in *PYD4* (Goldfarb et al., 2024). *AGT3*, on the other hand, remains uncharacterized.

We tested AGT2 enzymatic activity *in vitro* and we could not confirm that it can use β‐alanine as an amino donor (Figure S5). This remains an open question for future research.

Interestingly, it can also use l‐alanine as an amino donor, but only for degradation purposes. So far, AGT2 was not able to synthesize l‐alanine, unlike PYD4, which can. Although PYD4 can also use β‐alanine as an amino donor *in vitro*, its low expression in leaves and seeds, along with the lack of altered levels of β‐alanine in *pyd4* KOs, suggests that AGT2 is the primary enzyme involved in this reaction physiologically.

Compared to the wild type, the significantly increased levels of β‐alanine in *agt2* KO mutants, along with the significantly decreased levels of β‐alanine in the *AGT2* OE lines, support the hypothesis that AGT2 is involved in the degradation of β‐alanine. Furthermore, β‐alanine was the only metabolite that consistently changed across tissues/developmental stages in *agt2* KO (Figure S5). Moreover, *agt3* and *pyd4* KOs did not show any changes in β‐alanine levels in either leaves or seeds (Figure 3). These findings align with our previous phylogenetic analysis, where *PYD4* and *AGT3* clustered separately from the *AGT2* gene (Wu et al., 2016).

Notable accumulation of β‐alanine in *agt2* KO lines exerted overall changes in many primary and secondary metabolites and lipids (Figure 3). Coordination of metabolic components is of pivotal importance as they provide the building blocks and energy for cell division, expansion, resistance to stress, development, and growth. In Arabidopsis, previous studies from our lab proved that this metabolic coordination usually changes, shifts, or is lost when sudden non‐optimal conditions are applied or when a key metabolic enzyme is affected (Caldana et al., 2011; Fusari et al., 2017; Luzarowska et al., 2023). In addition to the increase in β‐alanine, *agt2* mutants showed stunted growth, reduced rosette diameter, and lower yield‐related traits (e.g., seed yield, seed viability and seed abortion) (Figures 4 and 5). Carbon availability and photoassimilate supply are directly related to plant growth parameters (Lauxmann et al., 2016; Sulpice et al., 2009). In addition, many metabolites and/or enzyme activities affecting primary metabolism have been linked to critical developmental processes such as germination, flowering, branching, and seed set (Fichtner et al., 2021; Fusari et al., 2017; Mason et al., 2014; Pracharoenwattana et al., 2010; Wahl et al., 2013). It has been previously shown that Arabidopsis ecotypes have high levels of *DIN1* (AT4G35770) and *BCAT‐2* (AT1G10070) transcripts after being subjected to a 32‐D condition (Luzarowska et al., 2023). It seems that the high accumulation of β‐alanine due to *agt2* mutations influences overall carbon levels and alters energy homeostasis, impacting several aspects of plant development. In plants, an important player coordinating energy sensing, the metabolic network, and the downstream physiological responses is the SUCROSE NON‐FERMENTING1 (SNF1)‐RELATED KINASE 1 (SnRK1). SnRK1 is a kinase complex harboring a catalytic α‐subunit and regulatory β‐ and γ‐subunits (Peixoto & Baena‐González, 2022). We found that the gene coding for the regulatory β‐subunit1 (*AKINBETA1*) is co‐expressed with *AGT2* and *ALDH6B2*. Under carbon starvation, SnRK1 activates catabolic processes and suppresses energy‐consuming processes. The stress condition tested here is subjecting the plants to a starvation scenario. Transcriptional studies determined that enzymes in the BCAA degradation pathways are activated by the SnRK1‐S1‐bZIP regulation pathway, therefore providing an alternative mitochondrial respiratory pathway crucial for plant survival in low energy environments (Pedrotti et al., 2018). In this context, our work sheds light on the regulatory role of *ALDH6B2* in controlling β‐alanine levels, as the final output of BCAA catabolism under stress conditions.

**Figure 6:**
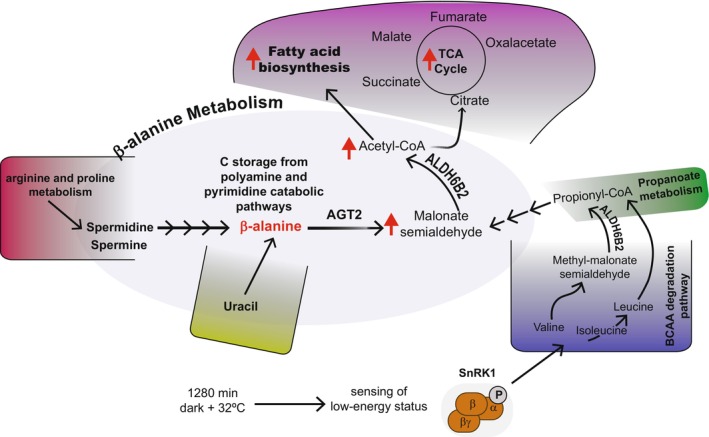
Key regulators of β‐alanine metabolism during starvation in Arabidopsis. In conditions that triggered starvation (e.g., 1280 min of dark +32°C), the SnRK1‐S1‐bZIP complex senses the low‐energy status and activates ALDH6B2, leading the production of malonate semialdehyde and Acetyl‐CoA. β‐alanine, which acts as a carbon (C) storage compound from the polyamine and pyrimidine catabolic pathways, is also key in the generation of malonate semialdehyde. This process redirects the carbon source to ATP‐ and NADPH‐generation pathways via Acetyl‐CoA synthesis, activating the TCA cycle, fatty acid synthesis, and/or β‐oxidation. AGT2 prevents the overaccumulation of β‐alanine, and dysfunction of this enzyme results in severe developmental defects and growth arrest.

Based on our current work and previous analysis (Wu et al., 2016) we propose that *AGT2* is a key gene in β‐alanine metabolism, particularly in β‐alanine degradation (Figure 6). Under carbon starvation, *ALDH6B2* and other enzymes in the BCAA catabolism are activated through the SnRK1‐S1‐bZIP regulatory pathway, inducing the generation of malonate semialdehyde. *ALDH6B2* is also important in the conversion of malonate semialdehyde to Acetyl‐CoA. Additionally, β‐alanine, which can be fueled through different pathways, serves as the reservoir of carbon in the synthesis of acetyl‐CoA for the TCA cycle, fatty acid synthesis, and/or β‐oxidation under low‐energy environmental conditions. *AGT2* then plays a critical role in directing the carbon source to the ATP‐ and NADPH‐generation pathways. This is supported by the fact that when *AGT2* is knocked out, acetyl‐CoA is abolished together with a massive deprivation of lipids, TCA cycle intermediates, and other stress‐related secondary metabolites such as flavonoids and some glucosinolates. In turn, many amino acids are accumulated as a result of an overaccumulation of β‐alanine (Figure 3). The role of *AGT2* in maintaining energy homeostasis and in redirecting carbon to energy generation processes seems to be crucial to avoid growth arrest and developmental problems, as evidenced by the dwarf and abortion phenotypes of *agt2* lines (Figures 4 and 5).

## SUMMARY AND CONCLUSIONS

GC–MS‐based metabolic GWAS provided new insights into the biological function of enzymes involved in key metabolite regulation in optimal and adverse environments. The current work unveiled *AGT2* function in plant development and growth, mostly due to its critical role in redirecting the carbon reservoir stored as β‐alanine to energy‐generation pathways, particularly in coordination with BCAA catabolism. Under environmental fluctuations, where carbon is scarce or the plant enters starvation, this carbon recirculation from β‐alanine to TCA cycle, fatty acid synthesis, and to the synthesis of secondary metabolites related to stress responses seems to be in coordination with SnRK1 signaling pathways sensing energy homeostasis. Evidence here also demonstrates that *AGT2* is involved in maintaining β‐alanine levels, if not in all plant organs. Finally, this work points out once more that central metabolism is a highly coordinated and regulated network that is controlled at multiple levels to assure plant growth and reproduction.

## EXPERIMENTAL PROCEDURES

### Plant materials

#### Natural population

A previously described diverse collection of 314 natural *A. thaliana* accessions, with existing SNP data, was used to measure primary metabolites for GWAS (Horton et al., 2012).

#### Knock‐out mutant lines

*A. thaliana* Col‐0 ecotype plants were used as control throughout the experiment. Two independent *agt2* knock‐out (KO) lines, SALK_003381 (*agt2_1*) and SALK_035035 (*agt2_2*), were previously used (Wu et al., 2016). In addition, we obtained six SALK lines and one SAIL line from the Arabidopsis Stock Center (Alonso et al., 2003) with T‐DNA insertions in *AGT3* (*AT2G38400*: SALK_100364, SALK_146687, and SALK_144859) and *PYD4* (*AT3G08860*: SAIL_400_G02, SALK_002102, SALK_001698, and SALK_141570). KO lines were selected on plates supplemented with kanamycin for SALK lines and BASTA for the SAIL line. The non‐segregating homozygous lines were then genotyped. The left primer (LP), right primer (RP) and border primer (BP) were designed using the Primer Design Tool provided by the Salk Institute Genomic Analysis Laboratory (http://signal.salk.edu/tdnaprimers.2.html) and were used in PCR to check the presence of the T‐DNA and zygosity in the offspring of the delivered seeds. Quantitative PCR analysis of the mutant lines was performed with gene‐specific primers. All the primers used in this study are shown in Table S18. All the T‐DNA insertion mutants were demonstrated to have completely knocked‐out expression of the gene.

### AGT2 cloning and overexpression

The full‐length coding sequence of *AGT2* was cloned from a cDNA amplified from 2‐week‐old Arabidopsis Col‐0 plants by PCR‐based Gateway BP cloning using the pDnor207 donor vector (Thermo Fisher Scientific, Waltham, MA, USA). Gene‐specific primers were used without the stop codon to ensure C‐terminal fusion of tags. The destination vector for overexpression was constructed using the Gateway LR reaction with pK7FWG2 (Karimi et al., 2002). *AGT2* cDNA was driven by the 35SCaMV (Cauliflower mosaic virus 35S) promoter. The transformation vectors were introduced into *Agrobacterium tumefaciens* strain GV3101 by electroporation, and the resulting strains were used to transform *A. thaliana* Col‐0 using the floral dip method (Clough & Bent, 1998). Seeds from three independent transformed plants were randomly named as *ATG2*‐OE1, *AGT2*‐OE2, and *AGT2*‐OE3 and used in the subsequent experiments.

### Experimental design

#### Stress experiment in the GWAS population

Seeds from Arabidopsis accessions were sown directly in soil in 6‐cm pots and stratified at long‐day with cold‐night condition (16 h long‐day photoperiod, 150 μmol m^2^ sec^−1^, day/night temperature of 20°C/6°C, relative humidity 60%/75%). After 2 weeks, seedlings were pricked to individual pots (*n* = 6 for each accession). Plants then grew in short days (8 h light) for another 2 weeks. Climate in the culture room was converted to long‐day condition (16 h light) for the next 2 weeks. Plants were placed randomly to avoid block effects during growth. All plants were watered daily for 5 min with 1/1000 Hyponex solution (Hyponex, Osaka, Japan), and the trays with plants were rotated horizontally every second day to prevent positional light effects.

To investigate the influence of abiotic stress on metabolome regulation, we chose (32°C + darkness) among all abiotic stresses previously tested in the time‐course experiment (Caldana et al., 2011). Then, we randomly divided six plant replicates for each accession into two equal groups: the ‘control’ condition (21‐L: 16 h long‐day photoperiod, 150 μmol m^−2^ sec^−1^, day/night temperature of 20°C/16°C and relative humidity 60%/75%), and the ‘stress’ condition (32‐D: 1280 continuous minutes of darkness at 32°C, relative humidity 75%), mimicking the previous experiment (Caldana et al., 2011). Harvesting of samples was done at 42 days post germination (dpg) for the control group and at 42 dpg plus 1280 min of stress group. All samples were collected randomly within a 1‐h time frame while exposed to light, to minimize any variation due to harvest. For each condition, three plants were pooled together to make one biological replicate of each accession, and frozen in liquid nitrogen. We followed a similar experimental design to that described in our recently published study (Luzarowska et al., 2023). All the samples were stored at −80°C until subsequent GC–MS metabolite profiling. Metabolite/gene expression correlation was done using previous data. Briefly, in addition to control (21‐L) and 32‐D (1280 continuous minutes of darkness at 32°C, relative humidity 75%), metabolites and transcripts were obtained from plants grown in another six stress conditions: 4‐D (1280 continuous minutes of darkness at 4°C), 4‐L (1280 continuous minutes of 150 μmol m^−2^ sec^−1^, at 4°C), 21‐D (1280 continuous minutes of darkness, at 21°C), 21‐LL (1280 continuous minutes of 75 μmol m^−2^ sec^−1^, at 21°C), 21‐HL (1280 continuous minutes of 400 μmol m^−2^ sec^−1^, at 21°C), 32‐L (1280 continuous minutes of 150 μmol m^−2^ sec^−1^, at 32°C) (Caldana et al., 2011).

#### Experiment using KO and OE lines

Seven different organs (seeds, seedlings, rosettes, flowers, stems, senescent leaves, and siliques) for *agt2* mutants and Col‐0 were harvested from six plants per line, as follows. Dry seeds from our previous study (Wu et al., 2016) were collected as is. Fresh seedlings: seeds from each line were pre‐sterilized and sown in separate plates containing 0.5× MS‐agar. After 2 days of stratification at 4°C, plates were placed in a growth chamber (16 h long‐day photoperiod, 150 μmol m^2^ sec^−1^, day/night temperature of 20°C/16°C and relative humidity 60%/75%). Seedlings were harvested after 10 days. Rosette leaves were acquired from 4‐week‐old plants. Flowers were collected from plants at anthesis. Stems, siliques, and ~90% senescent leaves were harvested from plants on the 47th dpg. All these organs were flash‐frozen in liquid nitrogen and stored at −80°C until subsequent GC–MS metabolite profiling. Plant cultivation was performed as described for the control group of GWAS population.

Rosette leaves for *agt2*, *agt3*, *pyd4* mutants, *AGT2*‐OE lines and Col‐0 were harvested from 4‐week‐old plants (*n* = 6 per line). Plant cultivation was performed as described for the control group of the GWAS population. All samples were flash‐frozen in liquid nitrogen and stored at −80°C until subsequent GC–MS metabolite profiling.

Dry seeds from six different plants were freshly collected from KO, OE, and Col‐0. Samples were flash‐frozen in liquid nitrogen and stored at −80°C until subsequent GC–MS metabolite profiling.

### Primary metabolite profiling by GC–MS

Metabolite extraction and derivatization from *A. thaliana* leaves using GC–MS were performed as described before (Lisec et al., 2006). The GC–MS data were obtained using an Agilent 7683 series auto‐sampler (Agilent Technologies Deutschland GmbH, D‐76337 Waldbronn, Germany; http://www.home.agilent.com), coupled to an Agilent 6890 gas chromatograph–Leco Pegasus two time‐of‐flight mass spectrometer (Leco Instrumente GmbH, 41069 Mönchengladbach, Germany; http://www.leco.com/). Identical chromatogram acquisition parameters were applied to those previously used (Caldana et al., 2011). Chromatograms were exported from the LECO CHROMATOF software (version 3.34) to the R software. Ion extraction, peak detection, retention time alignment, and library searching were obtained using the TargetSearch package from Bioconductor (Cuadros‐Inostroza et al., 2009). Day normalization and sample median normalization were conducted. The resulting data matrix was used for further analysis. We only included metabolites with non‐missing values across at least 50% of the samples measured, either for the GWA population or for the mutants and OE experiments. In all cases, metabolite intensities were log_2_‐transformed.

### Genome‐wide association studies

A compressed mixed linear model (MLM) (Zhang et al., 2010) was fitted using the Genome Association and Prediction Integrated Tool (GAPIT, Tang et al., 2016) package implemented in R. For metabolic GWA, log_2_ of intensity for b‐alanine, valine, leucine, or isoleucine across accessions was used as phenotypic data. For eQTL, expression values for each accession were calculated as 2^(−ΔΔCt)^ using either *RGS1‐HXK1 INTERACTING PROTEIN1/RHIP1* (AT4G26410) as the reference gene and used as phenotypic value. The 250 K SNPs from previous publications data (Horton et al., 2012) was set as the genotype. The MLM includes principal components as fixed effects to account for population structure (commonly called the ‘Q’ matrix) (Price et al., 2006) and a kinship matrix (commonly called the ‘K’ matrix) (Eu‐ahsunthornwattana et al., 2014) to account for family relatedness across the accessions. The SNP fraction parameter was set to 0.1 for covariates to avoid excessive computation, as recommended by the GAPIT. Other parameters were set to default values.

### Locus identification

The following procedure was applied to identify genomic regions associated with metabolite traits. First, the significant threshold of association was set to LOD value > log_10_(1 = *N*) (*N* is the number of SNPs used in the study) as described previously (Wen et al., 2015). The LOD threshold was 5.0 by using this method. The resulting SNPs with LOD ≥5.0 were then assigned to the same QTL if the genomic distance between them was less than 10 kb. Finally, all the genes within the resulting groups were taken as putative candidates.

### Epistasis analysis

To investigate epistatic effects between genes *AGT2* and *ALDH6B2*, we plot the average phenotypic value found in the GWAS population for the allelic combinations (haplotype) for SNPs m61274 and m160531 (lead SNPs in the association for *AGT2* and *ALDG6B2*, respectively). A two‐way anova was performed to test the interaction between m160531 and m61274.

### qRT‐PCR and eQTL analysis

RT‐qPCR was performed on three individual plants per accession. Each sample was tested in three technical replicates. RT‐qPCR was performed on an ABI Prism^®^ 7900 HT real‐time PCR system (Applied Biosystems/Life Technologies, Darmstadt, Germany) in 384‐well PCR plates with a total reaction volume of 5 μl (2 μl forward and reverse primer mixture, 0.5 μm each), 0.5 μl cDNA, and 2.5 μl Power SYBR^®^ Green‐PCR Master Mix (Applied Biosystems/Life Technologies) using the following cycling program: 50°C for 2 min, 95°C for 5 min, 40 cycles of 95°C for 15 sec and 60°C for 60 sec, followed by a denaturation step of 95°C for 15 sec, 60°C for 15 sec, followed by a continuous temperature increase (0.3°C sec^−1^) to 95°C (for 15 sec). Steps 5, 6, and 7 were introduced to record a dissociation or melting curve for each product in order to detect non‐specific amplification. Expression values for each accession were calculated as 2^(−ΔΔCt)^ using *RGS1‐HXK1 INTERACTING PROTEIN1/RHIP1* (AT4G26410) as the reference gene. Primers used are listed in Table S18.

### Network analysis

Pearson correlation coefficient (PCC) between transcripts and metabolites was calculated in R. PCC threshold for building edges between features (transcripts and metabolites) in networks was set to *P*‐value <0.05. Based on the PCC thresholds, undirected networks were constructed with nodes representing metabolite and transcript features, and edges connecting the nodes between features, with a PCC passing the threshold using the igraph package (Csárdi & Nepusz, 2006) in R.

### Pathway enrichment analysis

To identify and visualize the affected metabolic pathways in *agt2* KO plants in at least two organs, a proof‐of‐knowledge based Ingenuity Pathway Analysis (IPA) was performed with MetaboAnalyst (https://www.metaboanalyst.ca/MetaboAnalyst/home.xhtml). Firstly, relative levels for each differential metabolite were uploaded to the MetaboAnalyst platform, and relative peak areas were normalized by Pareto scaling. Next, compound ID associations were determined by matching to PubChem and KEGG. Thirdly, three parameters needed to be specified for pathway analysis: the pathway library, the algorithm for pathway enrichment analysis, and the algorithm for topological analysis. In this study, the *Arabidopsis thaliana* metabolite library was selected for further pathway enrichment and topological analysis. The other two parameters were set to default. Finally, pathway enrichment and pathway topological analysis were performed. The possible biological impacts of the perturbed pathways were evaluated by enrichment analysis.

### Statistics for KO and OE experiment

Metabolite intensity (log_2_‐transformed) was used for anova and *t*‐test in KO, OE, and Col‐0 plants, followed by correction for multiple comparisons using the ‘p.adjust’ function in R (http://www.r‐project.org/). Pairwise comparison was conducted by the Tukey HSD tests using ‘TukeyHSD’ function in R.

### Enzymatic assay

#### Synthesis and cloning of AGT2 and PYD4

Full‐length protein encoded by AT4G39660 (*AGT2*) weak and strong alleles have 476 amino acids. The first 29 amino acids contain the signal peptide; therefore, they were excluded from the sequence for the synthesis service. Similarly, the first 31 amino acids containing the signal peptide in *PYD4* were excluded for synthesis. *AGT2* and *PYD4* were codon optimized for bacterial expression in *E. coli*, and synthesis was performed by IDT (Integrated DNA Technologies). The genes were cloned into *Nde1* and *Xho1* sites of the pET24a plasmid (Merck Millipore) and fused to a Hisx6 tag at the C‐terminus.

#### Enzyme expression and purification

The plasmid pET24a::AT4G39660 and pET24a::AT3g08860 were transformed into *E. coli* BL21‐(DE3) (Merck Millipore). For protein expression and purification, the strains were grown in 1.0 L LB Broth containing 50 μg ml^−1^ kanamycin at 30°C to an OD600 nm of 0.45. Protein expression was induced by adding isopropyl β‐d‐1‐thiogalactopyranoside (IPTG) to a final concentration of 1 mm with continued growth at 18°C for 18 h. Cells were lysed by sonication after a 30‐min preincubation in 50 mm Tris PH8 buffer supplanted with 1 mg ml^−1^ lysozyme (Merck, Germany) and 500 U benzonase (Merck) on ice. The filtered lysate was purified by Ni‐NTA on a His‐TRAP FF 5‐ml column (GE Healthcare, USA) and dialyzed into 50 mm Tris pH 8.0 and 10% glycerol. Protein concentration was measured using the Bradford assay with bovine serum albumin as the standard (Bradford, 1976).

#### Enzyme activity measurement

The assay measured the production of amino acceptors facilitated by the removal of the amino group from the donor. l‐Alanine dehydrogenase (ADH) and pyruvate dehydrogenase (PDH) were used as coupling enzymes. We expected that PDH would mediate the conversion of NAD to NADH either from pyruvate or 3‐oxopropanoate. The assay for β‐alanine‐glyoxylate aminotransferase and β‐alanine‐2‐oxoglutarate aminotransferase consisted of varying amounts of β‐alanine, 50 μm glyoxylate/2‐oxoglutarate, and 3 μm of pure recombinant AT4G39660, 0.5 U ml^−1^ of pyruvate dehydrogenase, 50 μm CoA, and 0.2 mm NAD in 50 mm Tris (pH 8.0) to a final volume of 0.2 ml. The assay for β‐alanine‐pyruvate aminotransferase consisted of varying amounts of β‐alanine, 50 μm pyruvate, and 3 μm of pure recombinant At4g39660, 0.5 U ml^−1^ of l‐alanine dehydrogenase, 50 μm CoA, and 0.2 mm NAD in 50 mm Tris (pH 8.0) to a final volume of 0.2 ml.

The assay for l‐alanine aminotransferase consisted of varying amounts of l‐alanine, 50 μm glyoxylate/2‐oxoglutarate/pyruvate, 3 μm of pure recombinant AT4G39660, 0.5 U ml^−1^ of pyruvate dehydrogenase, 50 μm CoA, and 0.2 mm NAD in 50 mm Tris (pH 8.0) to a final volume of 0.2 ml. The reverse assay consisted of varying amounts of glycine/glutamate, 50 μm pyruvate, 3 μm l‐alanine dehydrogenase, 50 μm CoA, and 0.2 mm NAD in 50 mm Tris (pH 8.0) to a final volume of 0.2 ml.

The production of NADH was measured at A340in a Biotek Powerwave HT Synergy spectrophotometer. Both the forward and the reverse assays were incubated at 30°C. All kinetic constants were calculated using GraphPad Prism V5 software by using the nonlinear regression analysis algorithm feature.

Two types of no‐reaction controls were used: reaction without enzymes and reactions including only the coupling enzyme (alanine dehydrogenase, ADH, or pyruvate dehydrogenase, PDH). In all cases, the negative control did not show any changes in absorbance.

### Phenotypic analysis

#### Seed microscope analysis and seed abortion quantification

To quantify seed abortion, we randomly selected 40 siliques from five to six plants for each line (*agt2_1*, *agt2_2* and Col‐0), and checked the number of aborted and non‐aborted seeds in each silique by using a stereo microscope. We scored aborted seeds based on seed morphology.

#### Seed viability assays

To test seed viability, we incubated freshly dry seeds from *agt2_1*, *agt2_2*, and Col‐0 at 30°C in 1% aqueous tetrazolium solution (2,3,5‐triphenyltetrazolium chloride; Sigma‐Aldrich, https://www.sigmaaldrich.com) for 2 days in darkness (Verma & Majee, 2013). Between 50 and 80 seeds from three plants per line were analyzed. Seeds were mounted in water and examined under a microscope. Images of seeds were captured using a Leica Stereomicroscope MZ 12.5, DC DFC 420, Soft Imaging System (LAS) (Leica, http://www.leicabiosystems.com). In addition, freshly dry seeds from Col‐0 incubated 1 h at 100°C were used as the dead‐seed control. We classified seeds into three different groups based on their staining degree: viable seeds (dark brown/reddish), intermediately viable seeds (light brown) and dead seeds (yellowish).

#### Seed germination test and seedling growth

The obtained freshly‐dry *agt2* mutant and Col‐0 seeds were surface sterilized with 70% (vol/vol) ethanol for 1 min and 25% bleach for 10 min, followed by three washes with fresh water. The seeds were then sown in plates containing the growth media: Murashige and Skoog (MS) salts with 0.8% agar. To synchronize germination, seeds were stratified after sowing in a dark cold‐room (4°C) for 3 days and subsequently transferred to a growth chamber (23°C, 16 h‐light photoperiod). Germination rates were recorded daily for 10 days following stratification. Seeds were considered to have germinated when radicle emergence was visible under a dissecting microscope.

#### Seedling root experiment

The seeds from the *agt2* mutant lines and Col‐0 were sterilized as described above. For the seedling root experiment, the plates with the sown seeds were arranged vertically in racks after transferring to phytotrons with a 16‐h light photoperiod. The average light intensity was maintained at 150 μmol m^−2^ sec^−1^. The day/night temperatures were maintained at 20°C/16°C and 60%/75% relative humidity, respectively. The root length was measured and recorded for 10 days after stratification.

#### Rosette diameters and cell size

We monitored the rosette sizes (*n* = 4) of *agt2* mutants and Col‐0 from day 0 to 36 after emergence of the first true leaves. From them, we selected similar developmental stages and locations along the leaf lamina of three different leaves/rosettes to monitor cell size differences. The leaf tissue was treated with 12.5% acetic acid diluted in ethanol for 1 h. Then washed with 100% ethanol followed by 50% ethanol. After a final wash with water, the samples were suspended in 1 m KOH. The cleared tissue was mounted on a glass slide with a coverslip and observed on Olympus BX51. Cell size and rosette area were measured using Fiji (Schindelin et al., 2012).

#### Monitoring plant growth and development

The obtained freshly‐dry *agt2* and Col‐0 seeds were sown and cultivated as described for the GWA population: they were sown directly on soil in 6 cm pots and stratified at long‐day with cold‐night conditions (16 h LD, 150 μmol m^−2^ sec^−1^ at day/night temperature of 20°C/6°C, relative humidity 60%/75%). After 2 weeks, the seedlings were pricked to separate pots with seven replicates for each line. The plants grew thereafter in short days (8 h light) for another 2 weeks. The climate in the culture room was converted to long‐day conditions (16 h light) for the next 2 weeks. Plants were placed randomly to avoid block effects during growth. All plants were watered daily for 5 min with 1/1000 Hyponex solution (Hyponex, Osaka, Japan), and the trays with plants were rotated horizontally every two days in order to prevent positional light effects. The rosette size of each plant was recorded after 35 days' growth since the onset of germination. Plant height was recorded daily since the formation of floral buds in Col‐0.

#### Seed yields and weight per seed

The freshly dry seeds from *agt2* KO lines, *AGT2*‐OE lines, and Col‐0 were harvested from plants grown under exactly the same conditions of light, temperature, and humidity. The seeds from 10 plants of each line were accumulated and weighed, respectively. Weight per seed was obtained for five to six plants of each line, counting and weighing 15 to 100 seeds per plant per line. The large range of seeds weighed is mostly due to *agt2* KO mutants because they undergo abortion, and only viable seeds were taken to be weighed.

## AUTHOR CONTRIBUTIONS

SW performed most of the experiments and analysis; YZ, UL, and LY helped in growing, harvesting, and processing samples for GC–MS; LY and MAS helped with GC–MS analysis; VPT, NS, and AF helped discuss the experiments and their interpretation. AS assisted in microscopy analysis. SB designed and performed enzymatic assays. VEG ran LC–MS to determine substrate purity and products of enzymatic reactions. SW, CMF, and YB designed the study and wrote the manuscript. All authors helped to draft the manuscript and approved it.

## CONFLICT OF INTEREST

The authors declare there is no competing interest.

## Supporting information


**Data S1.** Metabolite levels (log_2_ normalized) for the complete mapping population grown under control conditions (21‐L: 21°C, 16‐h photoperiod, 150 μmol m^−2^ sec^−1^) or subjected to stress prior to harvesting (1280 min at 32°C in darkness). These data were used as phenotypes for conducting GWAS using the GAPIT software with its default parameter settings.

**Data S2.** *AGT2* transcript levels for the complete mapping population grown under control conditions (21‐L: 21°C, 16 h photoperiod 150 μmol m^−2^ sec^−1^) or subjected to stress prior to harvesting (1280 min at 32°C and darkness). Expression values for each ecotype were calculated as 2^(−ΔΔCt)^ using *RGS1‐HXK1 INTERACTING PROTEIN1/RHIP1* (AT4G26410) as the reference gene. These data were used as phenotypes for conducting GWAS using the GAPIT software with its default parameter settings.

**Data S3.** Sequences of *AGT2* strong and weak alleles, and *PYD4* reference allele used in the enzymatic assay.

**Figure S1.** GWAS for branched‐chain amino acids (BCAAs): valine, leucine, and isoleucine. Manhattan plots were obtained for valine, leucine, and isoleucine (log_2_ intensity) from plants grown under control conditions (21‐L: 21°C, 16‐h photoperiod, 150 μmol m^−2^ sec^−1^) or subjected to stress prior to harvesting (1280 min at 32°C in darkness). None of the BCAAs were associated either with *AGT2* or the *ALDH6B2* locus. Refer to Figure 1.

**Figure S2.** Epistasis between *AGT2* and *ALDH6B2*. Both loci are associated with β‐alanine in plants subjected to stress conditions (1280 min at 32°C + darkness) prior to harvest. The average metabolite level for accessions having the four possible combinations between the two alleles present in *AGT2* (A, C) and the two alleles present in *ALDH6B2* (A, T). A two‐way anova was performed to evaluate the interaction between the lead SNPs associated with either locus. The *P*‐value of interaction is stated in the figure. Refer to Figure 1.

**Figure S3.** Correlation between *AGT2* and 28 co‐expressed genes. Twenty‐eight genes co‐expressed with *AGT2* according to ATTED‐II and STRING databases were correlated using Caldana et al. (2011) time series of expression. Pearson coefficient of correlation is expressed as heatmap (reference in the figure). AGI code, number of significant correlations in parentheses, gene acronym, and operating pathways are included on the right. Asterisks highlight significant correlations (*P < 0.05, **P < 0.01, ***P < 0.001). Refer to Figure 2.

**Figure S4.** Heatmap of metabolic changes in rosettes and seeds for *pyd4* and *agt3* knock‐out (KO) lines. Changes in metabolic levels for *agt3* and *pyd4* were normalized to wild‐type levels (log_2_ fold change). Significant changes are marked with an asterisk (*P < 0.05). Intensity values of wild‐type Col‐0 and KO mutants are included in Tables S5 and S6. Refer to Figure 3.

**Figure S5.** AGT2 and PYD4 enzymatic assay. *In vitro* enzyme activity analysis using recombinant AGT2 (blue squares) and PYD4 (red circles). Transaminase activity was monitored by measuring NADH formation, coupled with either pyruvate dehydrogenase (a, b, e, g) or alanine dehydrogenase (c, d, f, h). NADH formation, represented as changes in absorbance at 340 nm, was plotted over time. Reactions tested β‐alanine or L‐alanine as amino donors, and pyruvate, glyoxylate, or 2‐oxoglutarate as amino acceptors. Initial reaction velocities (*V*_0_) were determined using the NADH extinction coefficient (*ε* = 6220 m ^−1^ cm^−1^ at 340 nm). Refer to Figure 3.

**Figure S6.** Primary metabolic changes in *agt2* knock‐out (KO) lines in different organs and developmental stages. (a) Venn diagram with number of metabolites detected commonly and specifically for each organ or developmental stage. (b) Venn diagram with number of metabolites that changed significantly compared to the wild type in different organs and developmental stages. (c) Heatmap of metabolic changes in *agt2* normalized to wild‐type values (log_2_ fold change, reference in the figure). Gray cells mean that the metabolite was not detected in the correspondent dataset and, therefore, normalization with the wild type was not possible. Significant changes are marked with an asterisk (*P < 0.05). Intensity values and statistics for wild‐type Col‐0 and KO mutants are included in Tables S9 and S10. Refer to Figure 3.

**Figure S7.** Pathway enrichment analysis with MetaboAnalyst. Metabolites significantly different in at least two organs from *agt2* KO mutants compared to wild‐type Col‐0 plants (see Figure S6) were uploaded to MetaboAnalyst for an Ingenuity Pathway Analysis (IPA). Pathway enrichment and pathway topological analysis were performed. The possible biological impacts of the perturbed pathways were evaluated by enrichment analysis. The matched pathways are displayed as circles. The color (reference in figure) and size of each circle were based on *P*‐value and pathway impact value, respectively. Refer to Figure 3.

**Figure S8.** Differential metabolic changes in primary metabolites, secondary metabolites, and lipids in *AGT2* overexpression lines and *agt2* knock‐out lines. Heatmap of metabolic changes (a: primary metabolites, b: secondary metabolites, c: lipids) in AGT2 OE lines and *agt2* KO lines, normalized to wild‐type values (log_2_ fold change, reference in the figure). Significant changes are marked with an asterisk (*P < 0.05). Refer to Figure 3.

**Figure S9.** Seed viability. Global snapshot of seed viability assay using tetrazolium solution. Col‐0 freshly harvested dry seeds were used as the positive control, while dead seeds, obtained by incubating Col‐0 freshly harvested dry seeds at 100°C for 1 h, served as the negative control. Seed viability is indicated by the intensity of the brownish color, with darker seeds indicating higher viability. A scale is included in the figure. Refer to Figure 4.

**Table S1.** Summary of amino acid changes for SNP variants in the β‐alanine mapped genes (AT4G39660 [*AGT2*] and AT2G14170 [*ALDH6B2*]). Refer to Figure 1.

**Table S2.** Summary of the 28 *AGT2*‐coexpressed genes and their annotation. Refer to Figure 2.

**Table S3.** Normalized primary metabolites from rosettes of *AGT2* overexpression lines and *agt2* knock‐out lines. Refer to Figure 3.

**Table S4.** Statistics for primary metabolites from rosettes of *AGT2* overexpression lines and *agt2* knock‐out lines. Refer to Figure 3.

**Table S5.** Normalized primary metabolites from rosettes and seeds of *agt3* and *pyd4* knock‐out lines. Refer to Figure 3.

**Table S6.** Statistics for primary metabolites from rosettes and seeds of *agt3* and *pyd4* knock‐out lines. Refer to Figure 3.

**Table S7.** Normalized primary metabolites, secondary metabolites, and lipid data in seeds of *AGT2* overexpression lines and *agt2* knock‐out lines. Refer to Figure 3.

**Table S8.** Statistics for primary metabolites, secondary metabolites, and lipids in seeds of *AGT2* overexpression lines and *agt2* knock‐out lines. Refer to Figure 3.

**Table S9.** Normalized primary metabolites in eight different organs of *agt2* knock‐out lines. Refer to Figure 3.

**Table S10.** Statistics for primary metabolites in different organs of *agt2* knock‐out lines. Refer to Figure 3.

**Table S11.** Accumulated germination rate for *AGT2* overexpression lines and *agt2* knock‐out lines. Refer to Figure 4.

**Table S12.** Quantification of aborted seeds in *AGT2* overexpression lines and *agt2* knock‐out lines. Refer to Figure 4.

**Table S13.** Quantification of seed viability in *agt2* knock‐out lines. Refer to Figure 4

**Table S14.** Root length in *agt2* knock‐out lines. Refer to Figure 4.

**Table S15.** Rosette diameter after emergence of first true leaves for *AGT2* overexpression lines and *agt2* knock‐out lines. Refer to Figure 5.

**Table S16.** Cell size in *agt2* knock‐out lines. Refer to Figure 5.

**Table S17.** Plant height after bud generation for *AGT2* overexpression lines and *agt2* knock‐out lines. Refer to Figure 5.

**Table S18.** Seed yield for *AGT2* overexpression lines and *agt2* knock‐out lines. Refer to Figure 5.

**Table S19.** Seed weight for *AGT2* overexpression lines and *agt2* knock‐out lines. Refer to Figure 5.

**Table S20.** Primers used in this study.

## Data Availability

Phenotypic data used for GWAS running (metabolite and transcript abundance) are available in Data S1 and S2, respectively. All data is available upon request to CMF (fusari@cefobi-conicet.gov.ar) and YB (brotmany@post.bgu.ac.il).
