# Supplementary material for: The homeostasis of β‐alanine is key for Arabidopsis reproductive growth and development
Source: Plant J. 2025 Apr 3;122(1):e70134. doi: 10.1111/tpj.70134 (PMC11969031; doi:10.1111/tpj.70134)
Supplement: Supplementary file 4 — Figure S1. GWAS for branched‐chain amino acids (BCAAs): valine, leucine, and isoleucine. Manhattan plots were obtained for valine, leucine, and isoleucine (log2 intensity) from plants grown under control conditions (21‐L: 21°C, 16‐h photoperiod, 150 μmol m−2 sec−1) or subjected to stress prior to harvesting (1280 min at 32°C in darkness). None of the BCAAs were associated either with AGT2 or the ALDH6B2 locus. Refer to Figure 1. [file TPJ-122-0-s023.pdf]

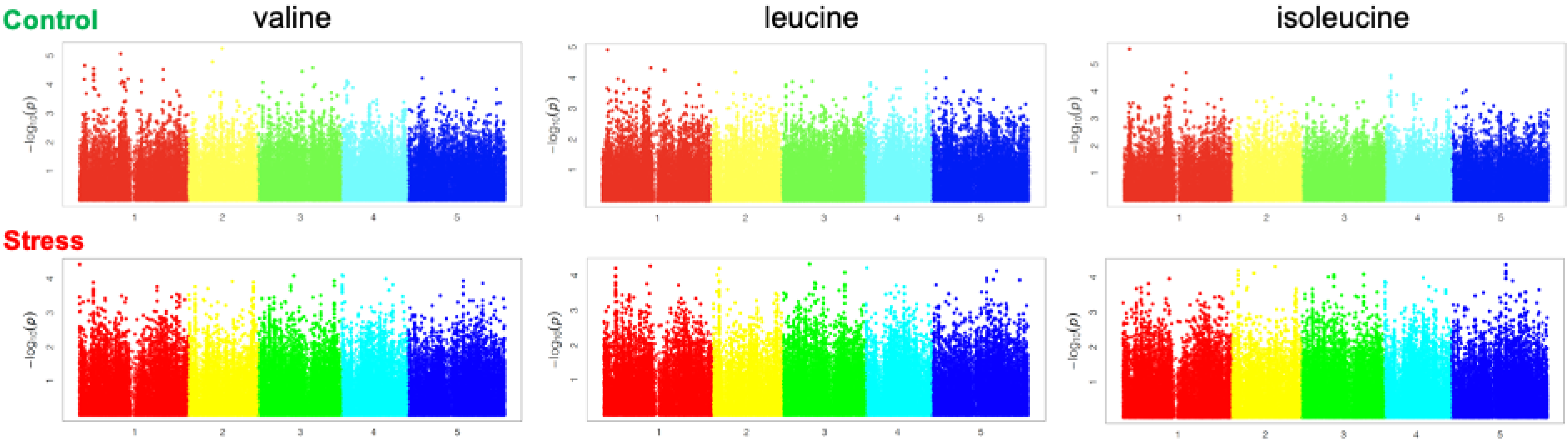

Figure S1. GWAS for branched-chain amino acids (BCAA): valine, leucine and isoleucine.
Manhattan plots were obtained for valine, leucine and isoleucine (log_2 intensity) from plants grown under control conditions (21-L: 21°C, 16-hour photoperiod, 150 µmol m^{-2} s^{-1}) or subjected to stress prior to harvesting (1280 minutes at 32°C in darkness). None of the BCAA were associated either with AGT2 or the ALDH6B2 locus. Refers to Figure 1.
