# Supplementary material for: The homeostasis of β‐alanine is key for Arabidopsis reproductive growth and development
Source: Plant J. 2025 Apr 3;122(1):e70134. doi: 10.1111/tpj.70134 (PMC11969031; doi:10.1111/tpj.70134)
Supplement: Supplementary file 5 — Figure S2. Epistasis between AGT2 and ALDH6B2. Both loci are associated with β‐alanine in plants subjected to stress conditions (1280 min at 32°C + darkness) prior to harvest. The average metabolite level for accessions having the four possible combinations between the two alleles present in AGT2 (A, C) and the two alleles present in ALDH6B2 (A, T). A two‐way anova was performed to evaluate the interaction between the lead SNPs associated with either locus. The P‐value of interaction is stated in the figure. Refer to Figure 1. [file TPJ-122-0-s005.pdf]

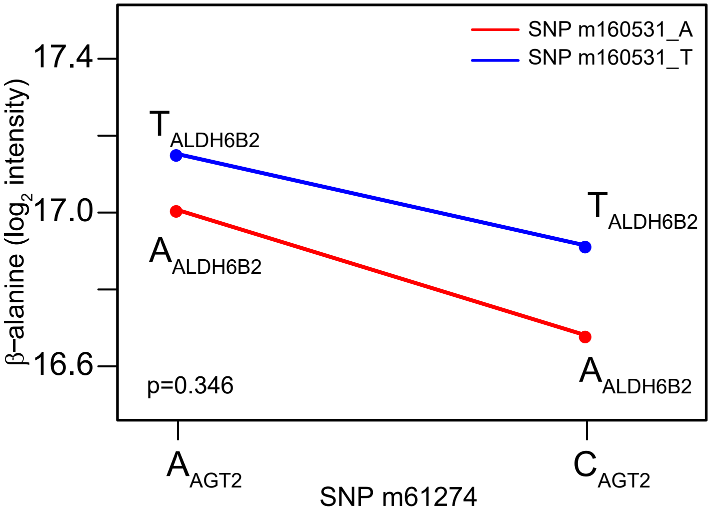

Figure S2. Epistasis between *AGT2* and *ALDH6B2.* Both loci associated with β-alanine in plants subjected to stress conditions (1280 min at 32ºC + darkness) prior to harvest. Average metabolite level for accessions having the four possible combinations between the two alleles present in AGT2 (A, C) and the two alleles present in ALDH6B2 (A, T). Two-way ANOVA was performed to evaluate the interaction between the lead SNPs associated to either locus. *P*-value of interaction is stated in the figure. Refers to Figure 1.
