# Supplementary material for: The homeostasis of β‐alanine is key for Arabidopsis reproductive growth and development
Source: Plant J. 2025 Apr 3;122(1):e70134. doi: 10.1111/tpj.70134 (PMC11969031; doi:10.1111/tpj.70134)
Supplement: Supplementary file 6 — Figure S3. Correlation between AGT2 and 28 co‐expressed genes. Twenty‐eight genes co‐expressed with AGT2 according to ATTED‐II and STRING databases were correlated using Caldana et al. (2011) time series of expression. Pearson coefficient of correlation is expressed as heatmap (reference in the figure). AGI code, number of significant correlations in parentheses, gene acronym, and operating pathways are included on the right. Asterisks highlight significant correlations (*P < 0.05, **P < 0.01, ***P < 0.001). Refer to Figure 2. [file TPJ-122-0-s011.pdf]

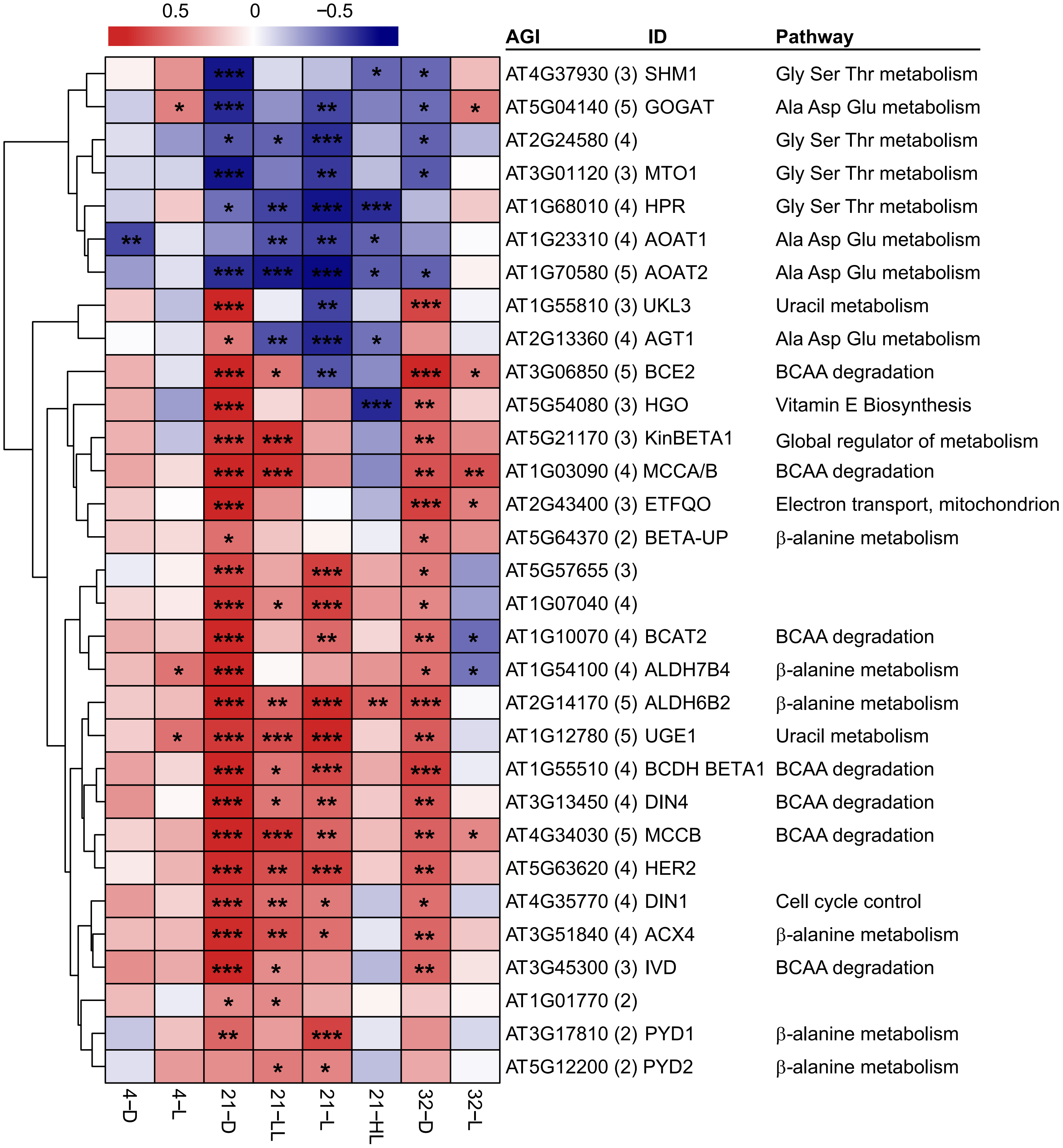


**Figure S3. Correlation between *AGT2* and 28 co-expressed genes.**
Twenty-eight genes co-expressed with *AGT2* according to ATTED-II and STRING data bases were correlated using Caldana et al. (2011) time-series of expression. Pearson coefficient of correlation is expressed as heatmap (reference in the figure). AGI code, number of significant correlations in parenthesis, gene acronym and operating pathways are included in the right. Asterisks highlight significant correlations (*$p$-value < 0.05, **$p$-value <0.01, ***$p$-value < 0.001). Refers to Figure 2.
