# Supplementary material for: The homeostasis of β‐alanine is key for Arabidopsis reproductive growth and development
Source: Plant J. 2025 Apr 3;122(1):e70134. doi: 10.1111/tpj.70134 (PMC11969031; doi:10.1111/tpj.70134)
Supplement: Supplementary file 7 — Figure S4. Heatmap of metabolic changes in rosettes and seeds for pyd4 and agt3 knock‐out (KO) lines. Changes in metabolic levels for agt3 and pyd4 were normalized to wild‐type levels (log2 fold change). Significant changes are marked with an asterisk (*P < 0.05). Intensity values of wild‐type Col‐0 and KO mutants are included in Tables S5 and S6. Refer to Figure 3. [file TPJ-122-0-s014.pdf]

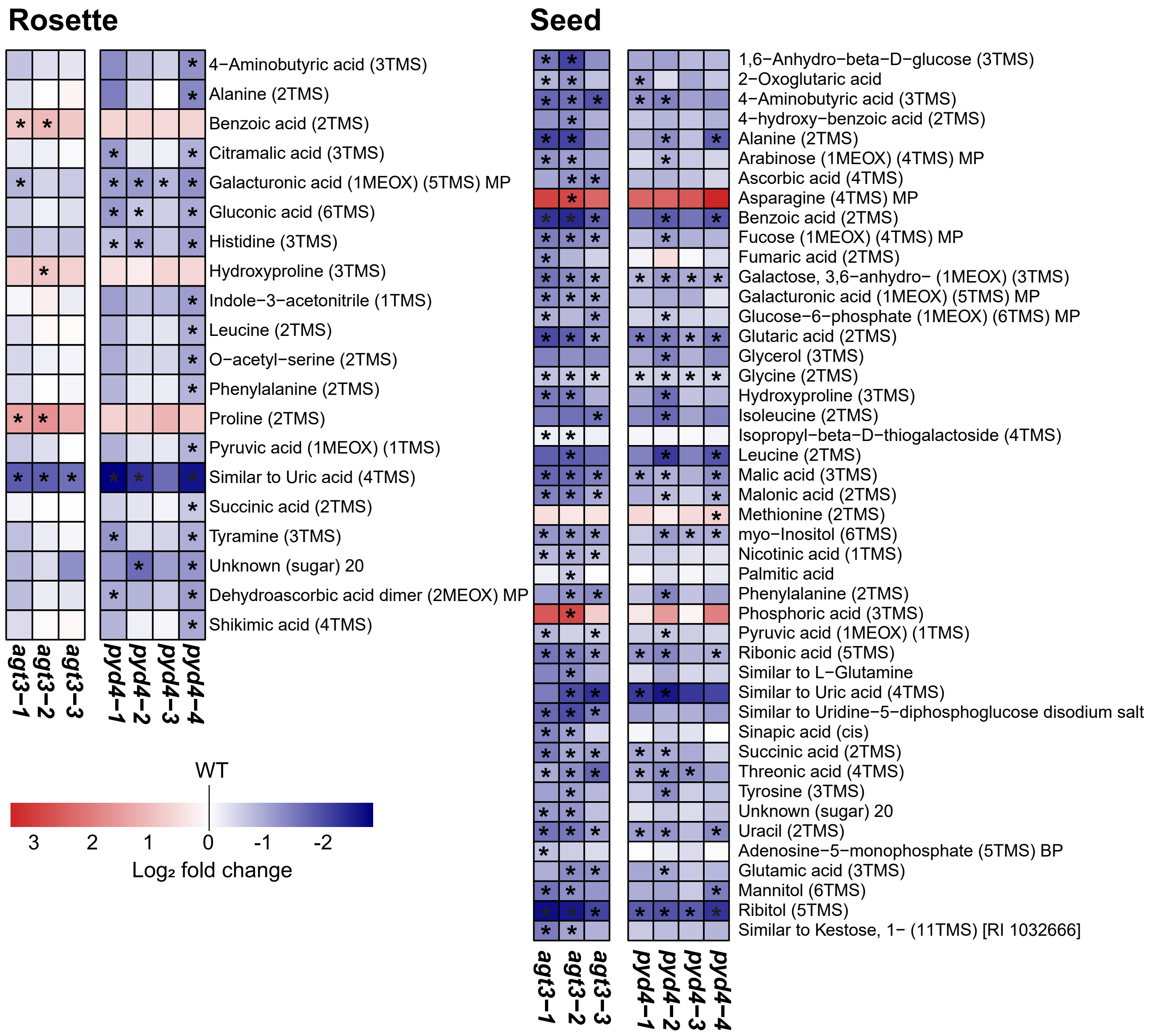

Figure S4. Heatmap of metabolic changes in rosettes and seeds of *agt3* and *pyd4* knock-out lines.
Changes in metabolic levels for *agt3* and *pyd4* were normalized to wild-type levels (log_2 fold change). Significant changes are marked with an asterisk (*p-value < 0.05). Intensity values or wild-type Col-0 and KO mutants are included in Table S5 and S6. Refers to Figure 3.
