# Supplementary material for: The homeostasis of β‐alanine is key for Arabidopsis reproductive growth and development
Source: Plant J. 2025 Apr 3;122(1):e70134. doi: 10.1111/tpj.70134 (PMC11969031; doi:10.1111/tpj.70134)
Supplement: Supplementary file 8 — Figure S5. AGT2 and PYD4 enzymatic assay. In vitro enzyme activity analysis using recombinant AGT2 (blue squares) and PYD4 (red circles). Transaminase activity was monitored by measuring NADH formation, coupled with either pyruvate dehydrogenase (a, b, e, g) or alanine dehydrogenase (c, d, f, h). NADH formation, represented as changes in absorbance at 340 nm, was plotted over time. Reactions tested β‐alanine or L‐alanine as amino donors, and pyruvate, glyoxylate, or 2‐oxoglutarate as amino acceptors. Initial reaction velocities (V 0) were determined using the NADH extinction coefficient (ε = 6220 m −1 cm−1 at 340 nm). Refer to Figure 3. [file TPJ-122-0-s006.pdf]

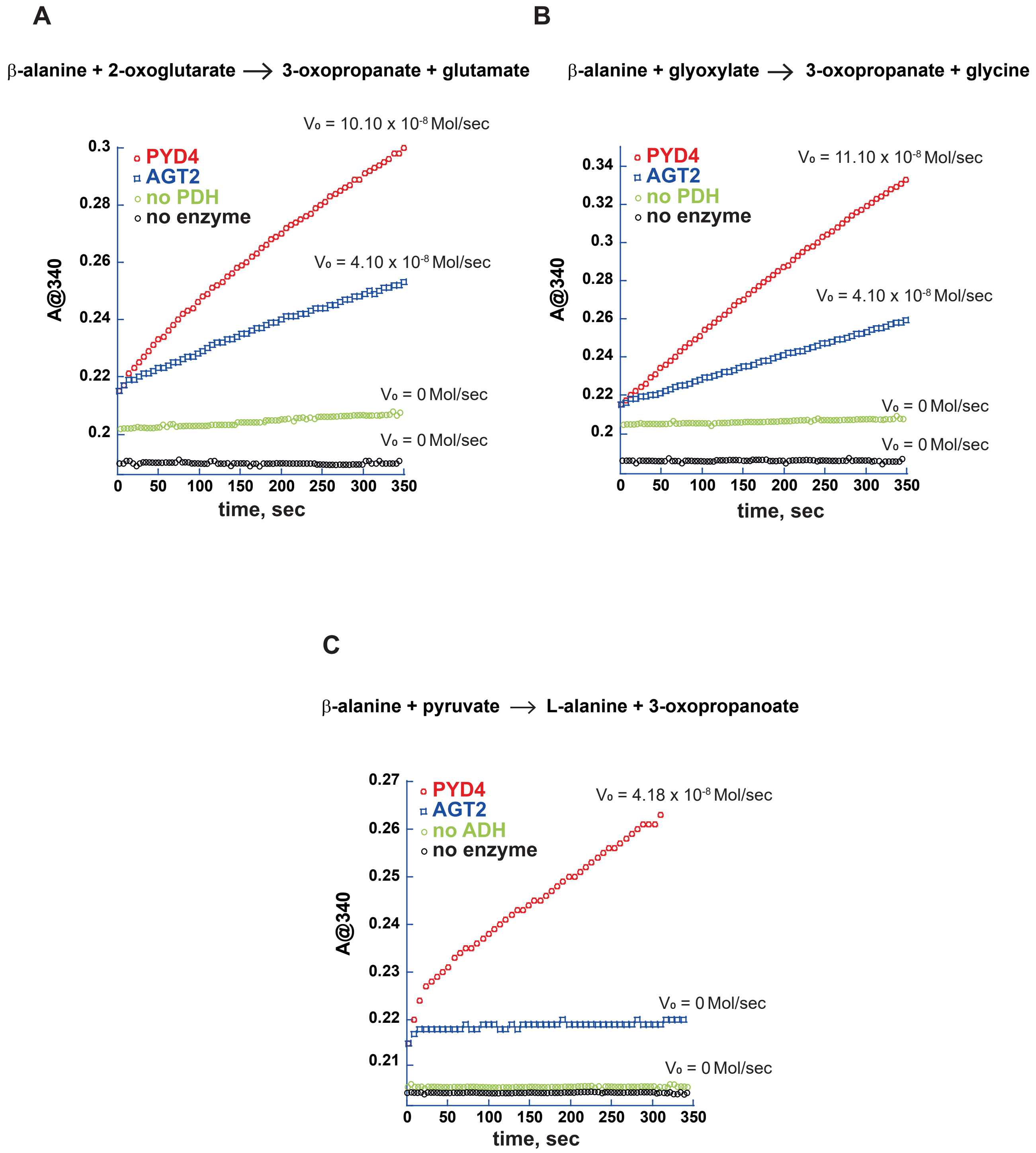
A
β-alanine + 2-oxoglutarate ⟶ 3-oxopropanate + glutamate
Vo = 10.10 x 10-8 Mol/sec
Vo = 4.10 x 10-8 Mol/sec
Vo = 0 Mol/sec
Vo = 0 Mol/sec
PYD4
AGT2
no PDH
no enzyme
A@340
0.3
0.28
0.26
0.24
0.22
0.2
0 50 100 150 200 250 300 350
time, sec
B
β-alanine + glyoxylate ⟶ 3-oxopropanate + glycine
Vo = 11.10 x 10-8 Mol/sec
Vo = 4.10 x 10-8 Mol/sec
Vo = 0 Mol/sec
Vo = 0 Mol/sec
PYD4
AGT2
no PDH
no enzyme
A@340
0.34
0.32
0.3
0.28
0.26
0.24
0.22
0.2
0 50 100 150 200 250 300 350
time, sec
C
β-alanine + pyruvate ⟶ L-alanine + 3-oxopropanoate
Vo = 4.18 x 10-8 Mol/sec
Vo = 0 Mol/sec
Vo = 0 Mol/sec
PYD4
AGT2
no ADH
no enzyme
A@340
0.27
0.26
0.25
0.24
0.23
0.22
0.21
0 50 100 150 200 250 300 350
time, sec

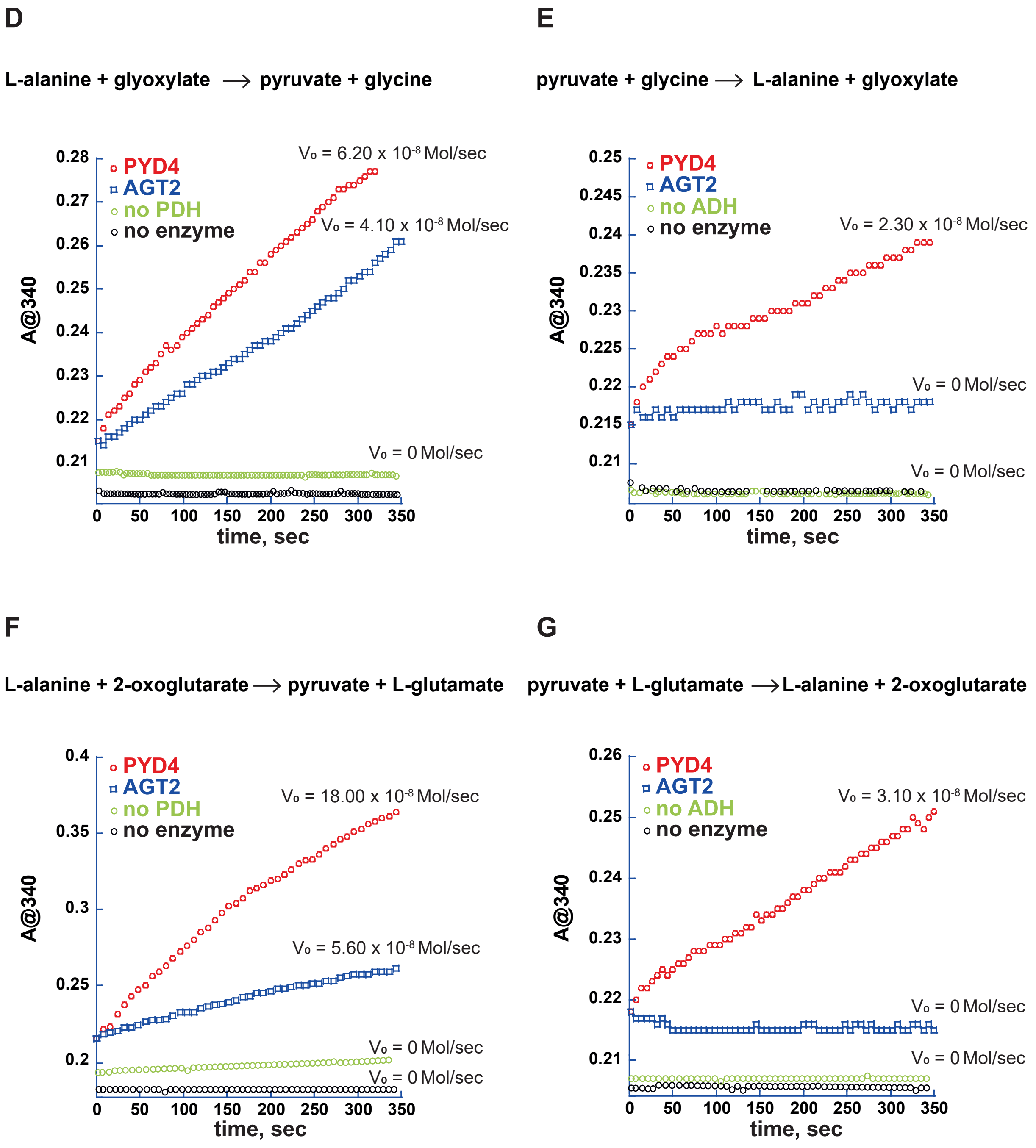

Figure S5. AGT2 and PYD4 enzymatic assay.
In-vitro enzyme activity using purified AGT2 (blue squares) or PYD4 (red circles). Two negative controls were included for each assay: no purified enzyme (black circles) and no coupling enzyme in the reaction (light-green circles). The transaminase activity was followed by NADH formation coupling pyruvate dehydrogenase (PDH) in A, B, D, F; or alanine dehydrogenase (ADH) in C, E, G. NADH formation, plotted as a function of time, is followed as a change in absorbance at 340 nm. Initial velocities of NADH production are calculated using NADH extinction coefficient at 340 nm of 6220 Mol-1 cm-1.
