# Supplementary material for: The homeostasis of β‐alanine is key for Arabidopsis reproductive growth and development
Source: Plant J. 2025 Apr 3;122(1):e70134. doi: 10.1111/tpj.70134 (PMC11969031; doi:10.1111/tpj.70134)
Supplement: Supplementary file 9 — Figure S6. Primary metabolic changes in agt2 knock‐out (KO) lines in different organs and developmental stages. (a) Venn diagram with number of metabolites detected commonly and specifically for each organ or developmental stage. (b) Venn diagram with number of metabolites that changed significantly compared to the wild type in different organs and developmental stages. (c) Heatmap of metabolic changes in agt2 normalized to wild‐type values (log2 fold change, reference in the figure). Gray cells mean that the metabolite was not detected in the correspondent dataset and, therefore, normalization with the wild type was not possible. Significant changes are marked with an asterisk (*P < 0.05). Intensity values and statistics for wild‐type Col‐0 and KO mutants are included in Tables S9 and S10. Refer to Figure 3. [file TPJ-122-0-s024.pdf]

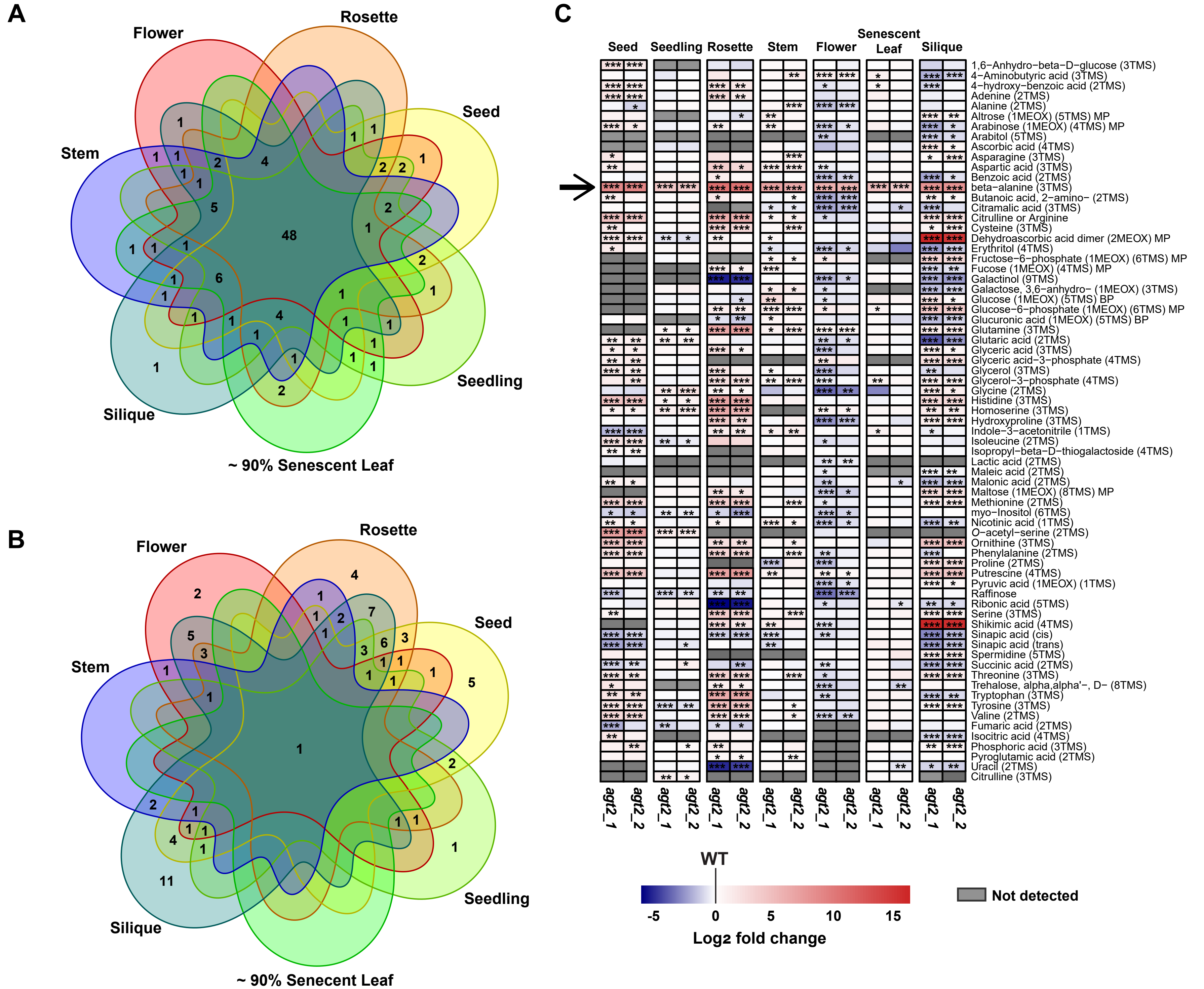

Figure S6. Primary metabolic changes in *agt2* knock-out lines in different organs and developmental stages.
A. Venn diagram with number of metabolites detected commonly and specifically for each organ or developmental stage. **B.** Venn diagram with number of metabolites that changed significantly compared to the wild type in different organs and developmental stages. **C.** Heatmap of metabolic changes in *agt2* KO normalized to wild-type values (log_2 fold change, reference in the figure). Black arrow highlights β-alanine position in the heatmap. Grey cells means that the metabolite was not detected in the corresponding dataset and, therefore, normalization with the wild type was not possible. Significant changes are marked with an asterisk (*p-value < 0.05). Refers to Figure 3.
