# Supplementary material for: The homeostasis of β‐alanine is key for Arabidopsis reproductive growth and development
Source: Plant J. 2025 Apr 3;122(1):e70134. doi: 10.1111/tpj.70134 (PMC11969031; doi:10.1111/tpj.70134)
Supplement: Supplementary file 10 — Figure S7. Pathway enrichment analysis with MetaboAnalyst. Metabolites significantly different in at least two organs from agt2 KO mutants compared to wild‐type Col‐0 plants (see Figure S6) were uploaded to MetaboAnalyst for an Ingenuity Pathway Analysis (IPA). Pathway enrichment and pathway topological analysis were performed. The possible biological impacts of the perturbed pathways were evaluated by enrichment analysis. The matched pathways are displayed as circles. The color (reference in figure) and size of each circle were based on P‐value and pathway impact value, respectively. Refer to Figure 3. [file TPJ-122-0-s027.pdf]

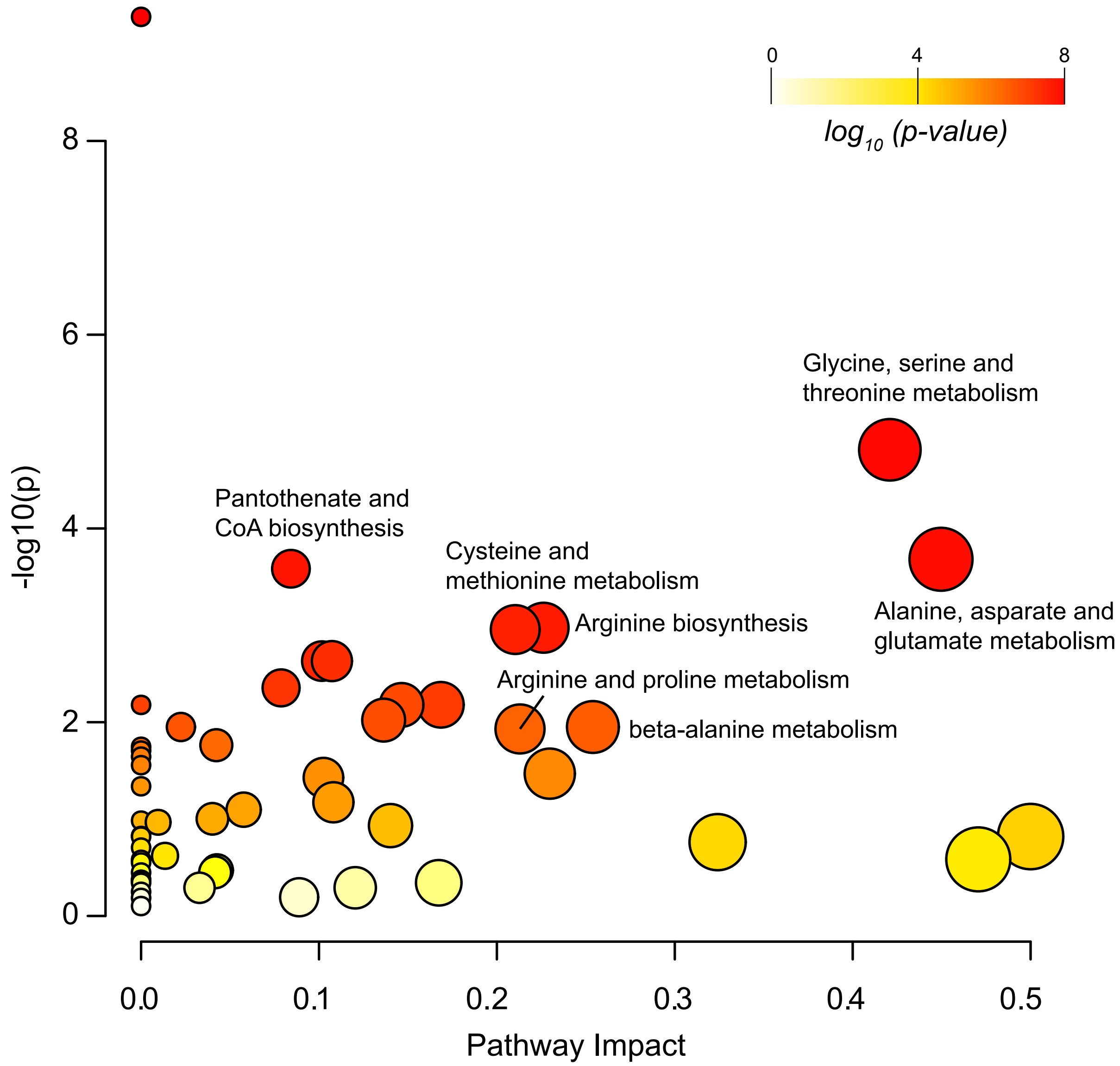


**Figure S7. Pathway enrichment analysis.**

Metabolites significantly different in at least two organs from *agt2* KO mutants compared to wild-type Col-0 plants (see Figure S6), were uploaded to MetaboAnalyst for a Ingenuity Pathway Analysis (IPA). Pathway enrichment and pathway topological analysis were performed. The possible biological impacts of the perturbed pathways were evaluated by enrichment analysis. The matched pathways are displayed as circles. The color (reference in figure) and size of each circle was based on *p*-value and pathway impact value, respectively. Refers to Figure 3.
