# Supplementary material for: The homeostasis of β‐alanine is key for Arabidopsis reproductive growth and development
Source: Plant J. 2025 Apr 3;122(1):e70134. doi: 10.1111/tpj.70134 (PMC11969031; doi:10.1111/tpj.70134)
Supplement: Supplementary file 11 — Figure S8. Differential metabolic changes in primary metabolites, secondary metabolites, and lipids in AGT2 overexpression lines and agt2 knock‐out lines. Heatmap of metabolic changes (a: primary metabolites, b: secondary metabolites, c: lipids) in AGT2 OE lines and agt2 KO lines, normalized to wild‐type values (log2 fold change, reference in the figure). Significant changes are marked with an asterisk (*P < 0.05). Refer to Figure 3. [file TPJ-122-0-s030.pdf]

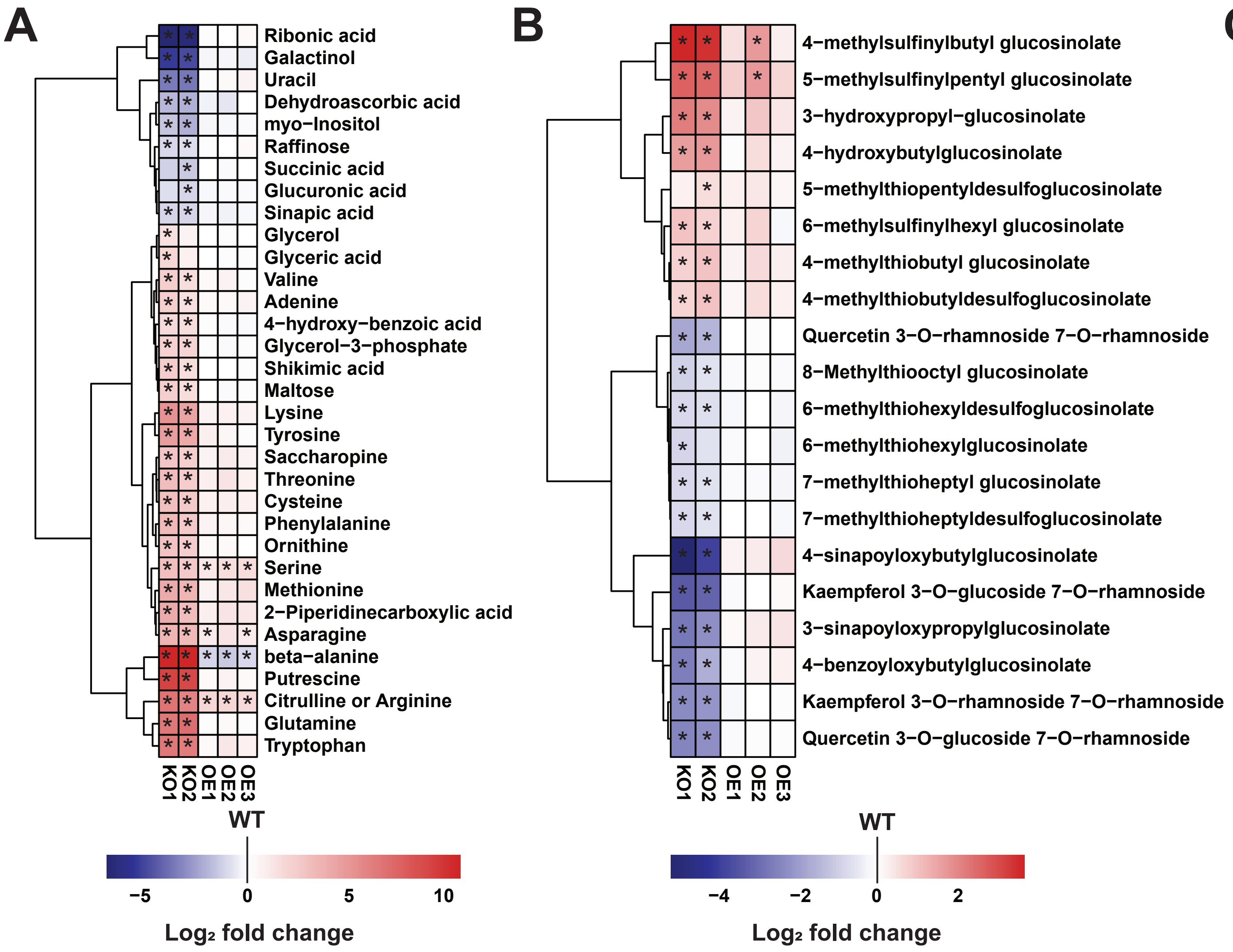

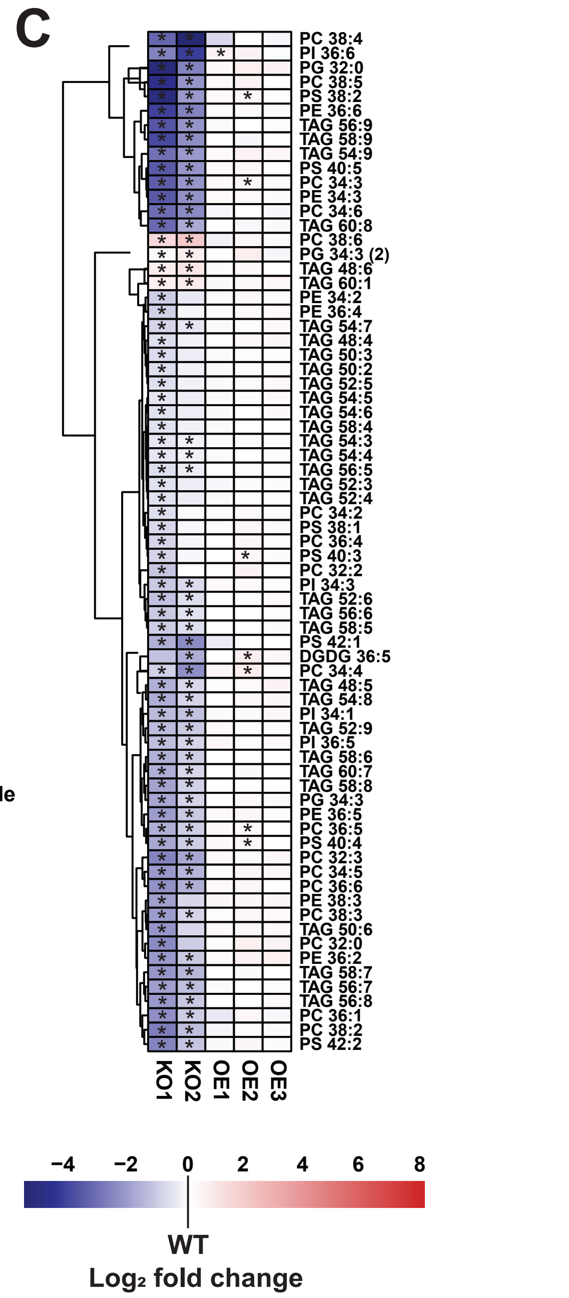

Figure S8. Differential metabolic changes in primary and secondary metabolites and lipids in *agt2* *KO* mutants and *AGT2* OE lines.

Heatmap of metabolic changes (**A**: primary metabolites, **B**: secondary metabolites, **C**: lipids) in *agt2* KO and *AGT2* OE lines normalized to wild-type values ($\log_2$ fold change, reference in the figure). Significant changes are marked with an asterisk (*p-value < 0.05). Refers to Figure 3.
