# Supplementary material for: The homeostasis of β‐alanine is key for Arabidopsis reproductive growth and development
Source: Plant J. 2025 Apr 3;122(1):e70134. doi: 10.1111/tpj.70134 (PMC11969031; doi:10.1111/tpj.70134)
Supplement: Supplementary file 12 — Figure S9. Seed viability. Global snapshot of seed viability assay using tetrazolium solution. Col‐0 freshly harvested dry seeds were used as the positive control, while dead seeds, obtained by incubating Col‐0 freshly harvested dry seeds at 100°C for 1 h, served as the negative control. Seed viability is indicated by the intensity of the brownish color, with darker seeds indicating higher viability. A scale is included in the figure. Refer to Figure 4. [file TPJ-122-0-s020.pdf]

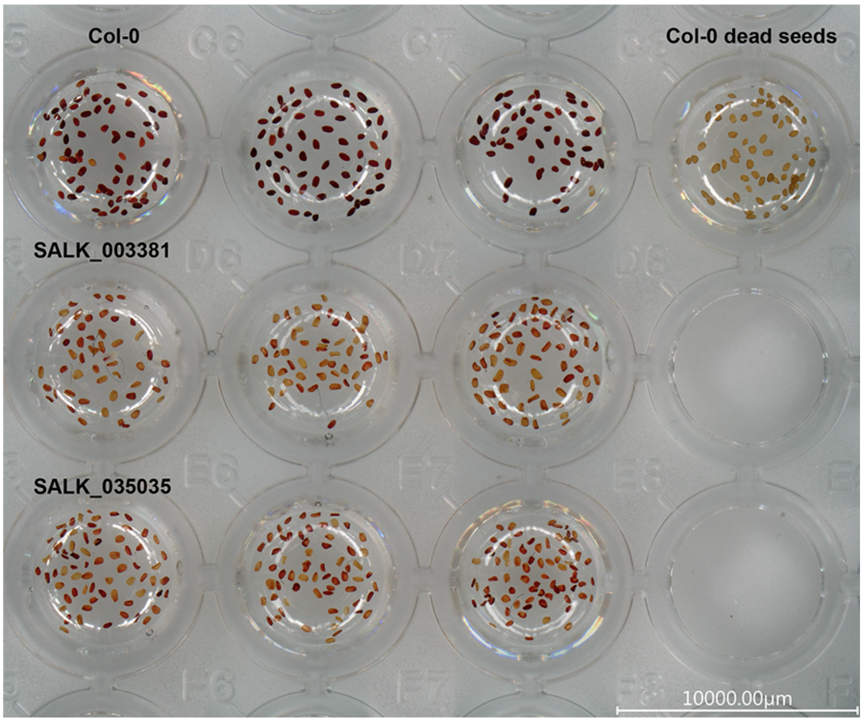

Figure S9. Seed viability.
Global snapshot of seed viability using tetrazolium solution. Col-0 freshly harvested dry seeds were used as positive control, while dead seeds, obtained by incubating Col-0 freshly harvested dry-seeds at 100ºC for 1 hour, served as the negative control. Seed viability is indicated by the intensity of the brownish color, with darker seeds indicating higher viability. Scale is included in the figure. Refers to Figure 4.
